# Altered behavior, brain structure, and neurometabolites in a rat model of autism-specific maternal autoantibody exposure

**DOI:** 10.1038/s41380-023-02020-3

**Published:** 2023-03-27

**Authors:** Matthew R. Bruce, Amalie C. M. Couch, Simone Grant, Janna McLellan, Katherine Ku, Christina Chang, Angelica Bachman, Matthew Matson, Robert F. Berman, Richard J. Maddock, Douglas Rowland, Eugene Kim, Matthew D. Ponzini, Danielle Harvey, Sandra L. Taylor, Anthony C. Vernon, Melissa D. Bauman, Judy Van de Water

**Affiliations:** 1 Department of Internal Medicine, Division of Rheumatology, Allergy, and Clinical Immunology, University of California, Davis, CA USA; 2 Department of Basic and Clinical Neuroscience, Institute of Psychiatry, Psychology and Neuroscience, King’s College London, London, UK; 3 Department of Psychiatry and Behavioral Sciences, University of California, Davis, CA USA; 4 Department of Neurological Surgery, University of California, Davis, CA USA; 5 MIND Institute, University of California, Davis, CA USA; 6 Center for Molecular and Genomic Imaging, University of California, Davis, CA USA; 7 Department of Neuroimaging, Institute of Psychiatry, Psychology and Neuroscience, King’s College London, London, UK; 8 Department of Public Health Sciences, University of California, Davis, CA USA; 9 MRC Centre for Neurodevelopmental Disorders, King’s College London, London, UK

**Keywords:** Autism spectrum disorders, Neuroscience

## Abstract

Maternal immune dysregulation is a prenatal risk factor for autism spectrum disorder (ASD). Importantly, a clinically relevant connection exists between inflammation and metabolic stress that can result in aberrant cytokine signaling and autoimmunity. In this study we examined the potential for maternal autoantibodies (aAbs) to disrupt metabolic signaling and induce neuroanatomical changes in the brains of exposed offspring. To accomplish this, we developed a model of maternal aAb exposure in rats based on the clinical phenomenon of maternal autoantibody-related ASD (MAR-ASD). Following confirmation of aAb production in rat dams and antigen-specific immunoglobulin G (IgG) transfer to offspring, we assessed offspring behavior and brain structure longitudinally. MAR-ASD rat offspring displayed a reduction in pup ultrasonic vocalizations and a pronounced deficit in social play behavior when allowed to freely interact with a novel partner. Additionally, longitudinal in vivo structural magnetic resonance imaging (sMRI) at postnatal day 30 (PND30) and PND70, conducted in a separate cohort of animals, revealed sex-specific differences in total and regional brain volume. Treatment-specific effects by region appeared to converge on midbrain and cerebellar structures in MAR-ASD offspring. Simultaneously, in vivo ^1^H magnetic resonance spectroscopy (^1^H-MRS) data were collected to examine brain metabolite levels in the medial prefrontal cortex. Results showed that MAR-ASD offspring displayed decreased levels of choline-containing compounds and glutathione, accompanied by increased taurine compared to control animals. Overall, we found that rats exposed to MAR-ASD aAbs present with alterations in behavior, brain structure, and neurometabolites; reminiscent of findings observed in clinical ASD.

## Introduction

Autism spectrum disorder (ASD) is clinically defined by a collection of symptoms including alterations in social, communicative, and stereotyped behaviors [1]. Despite these well-defined behaviorally diagnostic criteria, the molecular basis of these behaviors remains unclear. However, evidence exists for the dysregulation of important brain signaling molecules and pathways in ASD etiology; including imbalances in key neurotransmitters, gamma aminobutyric acid (GABA) and glutamate [2], or alterations in cellular metabolism including mitochondrial function [3] and one-carbon signaling [4]. Understanding which physiological systems and related signaling molecules are impacted, as well as the roles of developmental timing and biological sex on individual outcomes will aid in early diagnosis and therapeutic strategies in ASD.

Genetic influence is also an important factor in ASD, and studies investigating genetic risk have identified a multitude of candidate gene networks associated with diagnosis [5]. These include gene networks important for synaptic function [6], cellular metabolism [7], and immune signaling [8, 9]. The development of ASD, however, likely results from a combination of genetic and environmental factors, with early-life exposure to environmental insults playing an important role in some cases [10, 11]. In support of this view, numerous studies provide evidence that changes in maternal immune signaling during pregnancy associate with ASD diagnosis in offspring [12–15]. Prominent among these findings is that ~20% of mothers of children subsequently diagnosed with ASD possess circulating aAbs directed against proteins known to be important for early development. This phenomenon has been termed maternal autoantibody-related (MAR) ASD [16, 17]. We have previously shown that exposure to clinically relevant patterns of these aAbs results in changes to behavior, brain structure (as measured by sMRI), and neural progenitor cell proliferation in mice [18–20]. What remains to be understood are the effects of MAR-ASD aAb exposure on offspring across the lifespan, the impact of aAbs on cell signaling and target proteins, and the influence of sex as a biological variable.

Based on previous clinical findings suggesting larger total cerebral volume [21] in individuals from mothers with MAR-ASD aAbs, and recent data confirming that the presence of MAR-ASD aAb is positively correlated with behavioral outcomes [16], we hypothesized that offspring exposed to MAR-ASD aAbs would have an altered trajectory of brain growth and display changes in ASD-relevant behavioral outcomes. To test this, we created an endogenous model of MAR-ASD exposure in rats. The rat was chosen as a model system as laboratory rats have greater brain complexity and size, which begets enhanced cognitive ability, behavior, and suitability for neuroimaging studies compared to mice [22]. This builds upon our previous work [19], and provides an opportunity for greater translational relevance for the MAR-ASD model. Following model creation, offspring underwent longitudinal in vivo structural magnetic resonance imaging (sMRI) at postnatal day 30 (PND30) and PND70; with ages representing a pre-pubertal and post-pubertal time point, respectively. Additionally, a battery of behavioral tests was conducted across development to examine effects of aAb exposure on measures of communication, motor and reflex development, exploration, anxiety, sensorimotor gating, and social interactions. While collecting structural data during sMRI acquisition, we were also able to conduct magnetic resonance spectroscopy (MRS) to examine metabolite levels within a specific recording voxel placed in the frontal cortex of the rat offspring.

## Materials and methods

### Animals

Male and female Sprague Dawley rats were obtained from Charles River Laboratories (Portage, MI). All rats were pair-housed in a temperature- and humidity-controlled vivarium (22 ± 2 °C) on a 12:12 h light:dark cycle with food and water available ad libitum. All procedures were conducted in accordance with protocols approved by the University of California, Davis Institutional Animal Care and Use Committee. See Supplementary Methods for additional animal information.

After 2 weeks of acclimation, naïve rat dams were randomly assigned to treatment groups (Behavior: MAR-ASD; *N* = 5, Control; *N* = 6, Neuroimaging: MAR-ASD; *N* = 3, Control; *N* = 4). All animals then received subcutaneous immunizations, administered once weekly for a total of 4 weeks. The injection schedule and components are outlined in detail in Jones et al. [19] and only described briefly here. MAR-ASD rat dams received injections of 21 custom synthetic peptides corresponding to the immunodominant epitopes of LDH-A, LDH-B, STIP1, and CRMP1 (LifeTein LLC; Hillsborough, NJ). Peptide solutions, dissolved in sterile saline, were mixed with Freund’s complete adjuvant (CFA) for the first injection, or Freund’s incomplete adjuvant (IFA) for the three subsequent injections. Control dams were injected with adjuvant and saline only using the same injection schedule.

### Autoantibody confirmation

To verify aAb production to MAR-specific epitopes, blood was collected from rat dams 1 week after the final injection, on week 5, and centrifuged at 10,000 × *g* for 10 min. All blood draws and injections were conducted between the hours of 9 a.m.–12 p.m. Serum supernatant was then collected and stored a −80 °C until use. Dam and offspring blood samples were tested using an in-house enzyme-linked immunosorbent assay (ELISA). Results are expressed as fold change over baseline for aAb levels. Additional methodological details are in Supplementary Methods.

### Protein analysis

#### Multiplexed cytokine analysis

Serum collected from MAR-ASD rat dams for aAb confirmation was also used for cytokine analysis. Concentrations of cytokines and chemokines were determined using a commercially available multiplex bead-based kit according to manufacturer instructions (Bio-Plex Rat Cytokine 12-plex Assay; Bio-Rad Laboratories, Hercules, CA). Results were compared between MAR-ASD and control dams using two-way ANOVA analysis with Sidak’s testing for multiple comparisons.

#### Western blot

Brain tissue samples were collected from MAR-ASD and control offspring at PND2, and cell lysates were created. Approximately 50 mg of tissue was microdissected from the frontal pole and cerebellum of each animal and homogenized using a hand-held sonicator in 1X RIPA buffer (Thermo Fisher) with cOmplete^TM^ protease inhibitors (Roche) added. Samples underwent a freeze/thaw cycle, were spun to pellet cell debris, and supernatant was collected for protein analysis. Westerns were run in a mini-blot system (Thermo Fisher) using 12-well lane gels loading 15 ug of total protein into each well. Blots were then probed using secondaries against CRMP1 (Abcam; ab199722), STIP1 (Abcam; ab126724), or LDH (Abcam; ab52488). GAPDH and β-actin were used as loading controls for semi-quantitative measurement. Data were analyzed using Student’s *t* test.

#### Immunohistochemistry

Offspring underwent transcardial perfusion at PND2 with sterile saline and 4% paraformaldehyde to remove circulating factors and to fix the tissue. Brains were then collected and post-fixed overnight prior to embedding, freezing, and sectioning using a Leica cryostat. Coronal brain sections were placed on glass slides and immunostained for rat IgG (Jackson Immunoresearch; 112-547-003), DAPI (ThermoFisher; D1306), and NeuN (Abcam; ab177487). Representative images were taken using tiling with a 20x objective on a Leica TCS SP8 confocal microscope.

### Longitudinal behavioral testing

A summary of behavioral tests and testing ages in postnatal days (PND) are provided in Table 1. Protocols are described in detail in supplemental methods. For behavioral studies, animals were tattooed for identification purposes and litters culled to 8 (4 males/4 females). For the pre-weaning milestones and isolation-induced pup ultrasonic vocalizations (USVs), the entire litter was tested (6 MAR-ASD dams (*N* = 23M/25F); 5 control dams (*N* = 20M/20F)). For all other behavioral experiments, 2 male and 2 female offspring from each dam (6 MAR-ASD dams (*N* = 12M/12F); 5 control dams (*N* = 10M/10F)) were selected at random following weaning at PND 21 to participate in the behavioral test battery.

**Table 1:** Summary of behavioral tests used and testing ages in the postnatal period.

**Pre-weaning behavioral tests**	**Postnatal day (PND)**		**Description**
Developmental milestones	4,8,12		Screen for developmental delays (motor, reflex, etc.)
Isolation USVs	4,8,12		USVs in response to temporary separation
**Post-weaning behavioral tests**	**Juvenile (PND)**	**Adult (PND)**	
Elevated plus maze (EPM)	26	96	Anxiety related behaviors
Open field	29	100	Exploratory locomotion
Pre-pulse inhibition (PPI)	34	102	Sensory gating
Three-chamber sociability and social novelty test	28	98	Automated assessment of sociability and preference for novel versus familiar social partners
Social dyads	36	55 (Young adult) 103 (Adult)	Analysis of 10 min reciprocal social behavior with a novel partner at three developmental timepoints

### Magnetic resonance imaging and spectroscopy

For sMRI and ^1^H-MRS experiments, a total of 2 male and 2 female offspring from each dam (MAR-ASD (*N* = 6 M/6 F); Control (*N* = 8 M/8 F)) were selected at random. Dams in this cohort were bred specifically for neuroimaging analysis and did not overlap with those used for behavior. The sMRI and ^1^H-MRS data were acquired from these animals in vivo and longitudinally at PND30 and PND70. Specifics on MR acquisition, processing, and analysis can be found in the Supplementary Methods.

### Statistical analyses

For details on statistical analysis of behavioral and sMRI/S data, please see the Supplementary Materials.

## Results

### MAR-ASD aAb production is stable and does not induce persistent inflammation

**Fig. 1:**
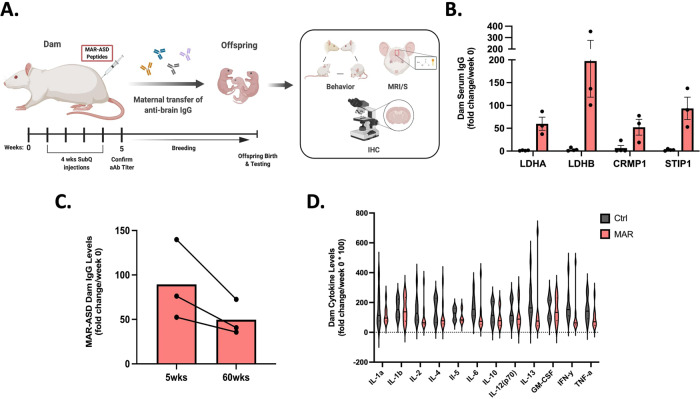
MAR-ASD autoantibody production in rat dams. **A** Autoantibody production is induced in rat dams through injection with autism-specific autoantigen peptides. Antibody can then transfer to offspring and influence developmental outcomes. **B** Levels of serum IgG Ab reactive to MAR-ASD proteins (LDHA/B, STIP1, CRMP1) in rat dams compared between aAb and control animals. Values expressed as fold-change over baseline (week 0) following 4 weeks of injections. **C** Longitudinal characterization of MAR-ASD IgG persistence in serum from treated dams at 5 weeks and 60 weeks following initial immunization. ELISA data representative of single experiments. **D** Serum cytokine levels in MAR-ASD and control dams taken 1 week prior to breeding. Data expressed as fold change over baseline (week 0). *N* = 3 per condition for each experiment, data are expressed as mean ± SEM. Analysis conducted by two-way ANOVA, representative of a single experiment.

We previously demonstrated the ability to induce aAb production in mouse dams to MAR-ASD-specific antigens [19]. Herein, we replicate and expand upon these findings in a rat model. To generate the model, rat dams were injected subcutaneously with an emulsion of MAR-ASD-specific peptide epitopes from a core, clinically-associated protein pattern (lactate dehydrogenase A (LDHA) and B (LDHB), collapsin response mediator protein 1 (CRMP1) and stress-induced phosphoprotein 1 (STIP1); [16, 17]) dissolved in sterile saline and mixed at a 1:1 ratio with Freund’s Adjuvant. Control animals were injected with adjuvant and saline alone at the same ratio. Injections were performed once weekly over a span of 4 weeks. After confirming the presence/absence of MAR-ASD aAbs, dams were bred to untreated sires and offspring outcomes were assessed using behavioral testing, in vivo longitudinal neuroimaging, and post-mortem analysis (Fig. 1A).

To verify aAb responses in rat dams, we developed an in-house enzyme linked immunosorbent assay (ELISA) to detect antibody (Ab) reactivity to LDHA, LDHB, CRMP1 and STIP1 in rat sera. Analysis of dam sera 5 weeks after the initial injection demonstrated robust Ab responses to MAR-ASD autoantigens in female rats (Fig. 1B). Moreover, these responses remained remarkably persistent across the lifespan of the animal, without the need for subsequent boosting (Fig. 1C). Characterization of the aAb response in rat dams revealed that antigen-specific Ab isotype and subclass followed a species-typical distribution for rats, albeit with some variation by antigen (Fig. S1A; [23]). Following confirmation of this specific aAb response present only in dams exposed to MAR-ASD autoantigens, we sought to clarify the impact of aAbs in this model by ruling out a role for active inflammation in observed outcomes, as is seen in other immune-related ASD models such as maternal immune activation (MIA) [24]. Therefore, we conducted a thorough analysis of serum cytokine and chemokine levels using a multiplexed Luminex assay on rat dam samples taken 1 week prior to breeding. Analysis of results confirmed that the levels of circulating cytokines in MAR-ASD dams did not differ from control animals (Fig. 1D). Cumulatively, these results suggest that MAR-ASD aAb responses were robust, long-lasting, and any effects on MAR-ASD offspring are not the result of a pro-inflammatory environment at the time of breeding.

### Transfer of aAbs to offspring and effects on target protein expression

**Fig. 2:**
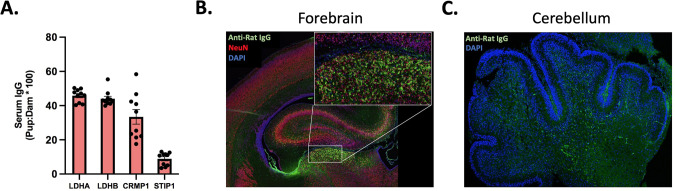
Transfer of MAR-ASD aAb to offspring. **A** Levels of serum IgG reactive to MAR ASD protein targets in exposed offspring at PND2. Data represent offspring ELISA reactivity expressed as a percentage of dam levels. *N* = 10 pups, representative of a single experiment with data expressed as mean ± SEM. Representative IHC images of IgG reactivity in the brain of PND2 MAR-ASD offspring in both the Forebrain (**B**) and Cerebellum (**C**). Tissue labeled and imaged across multiple experiments. Magnification ×20 objective.

To understand if MAR-ASD aAbs may impact offspring by entering the fetal compartment and interacting with target proteins there, we first examined the sera of neonatal offspring (PND2) for aAb reactivity by ELISA. We observed that antigen-specific MAR-ASD aAbs were present in offspring circulation at appreciable quantities. IgG reactive to LDHA, LDHB, and CRMP1 was present at ~30–50% of the observed maternal concentration, while Abs to STIP1 were present at a slightly lower level (Figs. 2A and S1B). Given that aAbs were able to gain access to offspring circulation, we next wanted to examine whether deposition of Ab could be observed in discrete tissues. Since ASD is a neurodevelopmental disorder and several MAR-ASD aAb targets, namely STIP1 and CRMP-family proteins, are upregulated during processes such as neural progenitor cell induction [25, 26], we examined Ab deposition in the brains of exposed offspring. Immunohistochemical (IHC) analysis of brain tissue from PND2 offspring revealed IgG deposition in the forebrain and cerebellum of MAR-ASD aAb exposed rat pups (Fig. 2B, C). Furthermore, IgG reactivity displayed a regional distribution and was enriched in NeuN+ positive cells in the forebrain indicating a preference for IgG binding to post-mitotic neurons (Fig. 2B).

To understand if IgG present in the brain may exert effects on target protein expression, we conducted western blot (WB) analysis of regional brain lysates from PND2 offspring to examine whether the level of STIP1, CRMP1, or LDH protein is altered in response to MAR-ASD aAb exposure. In both the frontal region of the brain, as well as in the cerebellum, it appeared that the levels of MAR-ASD aAb target proteins (STIP1, CRMP1, and LDH) were not significantly altered in response to MAR-ASD aAb exposure, as no statistical differences were observed between treatment groups using our methods (Fig. S1C–F). Together, these results suggest that MAR-ASD aAbs produced in treated dams can cross into offspring circulation and potentially access the brain.

### MAR-ASD aAb exposure results in behavioral deficits in offspring

Changes in species-typical social behavior have emerged as a feature of previous MAR-ASD mouse and non-human primate models [19, 27]. Therefore, we assessed longitudinal social development in exposed offspring using the three-chambered social approach and novelty task, as well as an unconstrained social dyad test. Additional behavioral assays conducted include evaluation of developmental milestones, isolation-induced pup ultrasonic vocalizations (USVs), elevated-plus maze (EPM), open field, and pre-pulse inhibition (PPI). For brevity, only significant behavioral differences in MAR-ASD offspring are presented in detail.

**Fig. 3:**
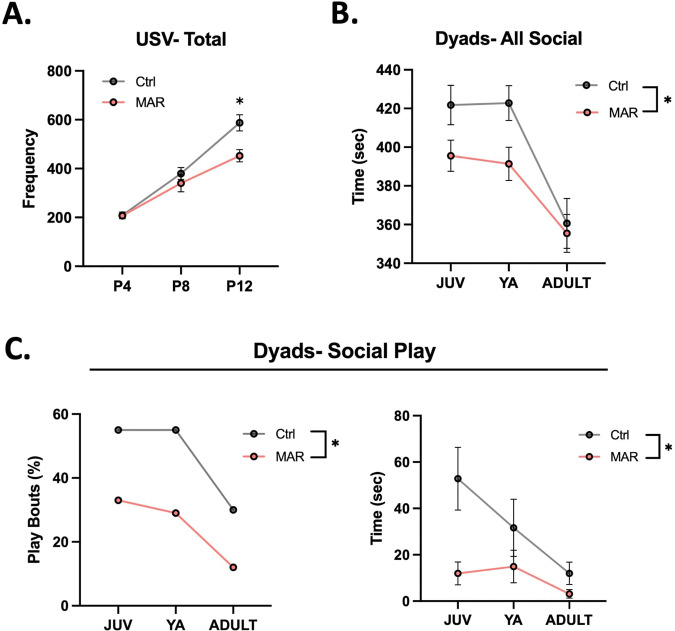
MAR-ASD aAb exposure alters offspring behavior. **A** Total ultrasonic vocalizations (USVs) recorded at each timepoint, graphed longitudinally by treatment (MAR; *N* = 48, Ctrl; *N* = 40). **B** Time spent engaged in social behavior during the social dyads task, measured in seconds and compared between MAR-ASD (MAR) and control (Ctrl) animals. Data are expressed as mean ± SEM, *p < 0.05. **C** Proportion of social play bouts by treatment (MAR; JUV (0.33, 95% CI’s = 0.16, 0.55), YA (0.33, 95% CI’s = 016, 0.55), ADULT (0.12, 95% CI’s = 0.03, 0.32), Ctrl; JUV (0.55, 95% CI’s = 0.31, 0.77), YA (0.55, 95% CI’s = 0.31, 0.77), ADULT (0.3, 95% CI’s = 0.12, 0.54) as well as total time spent engaged in social play behavior. Data expressed as mean ± SEM, *p < 0.05. Data expressed as mean ± SEM, *p < 0.05. MAR; *N* = 24; Ctrl; *N* = 20 for **B**–**C**. Behavioral data analyzed using linear mixed effects models with data collected across multiple experiments.

We found that offspring from MAR-ASD rat dams exhibited behavioral alterations across multiple domains including differences in communicative and social behavior. Specifically, we found that MAR-ASD rat offspring produced significantly fewer bouts of maternal-isolation induced USVs at PND12 compared to control offspring (*p* = 0.006; Fig. 3A). Analysis of developmental milestones in these animals revealed that MAR-ASD rat offspring also had increased negative geotaxis (*p* = 0.014; Fig. S2A) and lower body temperature (*p* = 0.017; Fig. S2B) at PND 12, suggesting a potential impact of MAR-ASD exposure on directional reorienting behavior, but not in cliff avoidance or righting reflexes at that timepoint. No sex differences were observed in response to treatment in either USV outcomes or developmental milestones (Fig. S2C and Table S1). MAR-ASD rat offspring also differed from controls throughout development when allowed to freely interact with a novel partner in an unconstrained social dyad paradigm. Results revealed that MAR-ASD rat offspring spent significantly less time engaging in all social interaction behaviors compared to controls (*p* = 0.023, Fig. 3B). More specifically, MAR-ASD rats were less likely to engage in social play behavior than control animals and spent less time playing when they did engage in such behavior (*p* = 0.010, Fig. 3C). No sex differences were seen in social play behavior in response to treatment (Fig. S2D). Although not statistically significant, the two-part model suggested potential group differences in self-grooming behavior (*p* = 0.078), warranting further investigation. No treatment differences were observed in automated assessments of sociability using the three-chamber sociability and social novelty test (Fig. S3B) or in performance on the elevated-plus maze, open field, or pre-pulse inhibition test (Table S1 and Fig. S4).

### Sex-specific neuroanatomical alterations in MAR-ASD offspring

Data from clinical studies of individuals with ASD suggest differences in both total brain volume (TBV; e.g., macro- or microcephaly; [28–30]) and at the regional level across a range of areas from the cerebellum to the frontal lobe, as measured by sMRI [31, 32]. Therefore, we characterized total and regional brain volumes in MAR-ASD exposed and control rat offspring using longitudinal in vivo *s*MRI acquired at PND30 and PND70, to understand whether MAR-ASD aAb exposure results in neuroanatomical differences to better understand the pathobiology of this model.

**Fig. 4:**
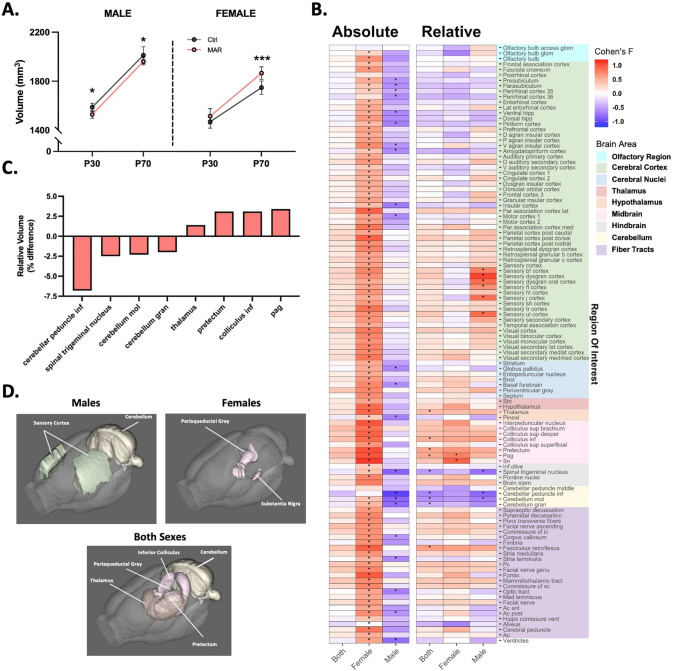
Altered regional brain volume in MAR-ASD offspring. **A** Total brain volume (mm^3^) in MAR-ASD and Ctrl offspring in both sexes at PND30 and PND70. Data represented as mean ± SD, *p < 0.05, ***p < 0.001. MAR ASD; *N* = 6/sex, Ctrl; *N* = 8/sex. **B** Heatmap plot of absolute and relative regional volumetric differences in MAR-ASD offspring collapsed between time points. Heatmap scale corresponds to Cohen’s *F* values for the comparisons between MAR-ASD and Ctrl animals for each region. Columns represent data either from both sexes combined (“Both”), or each sex independently. Starred (*) fields represent those comparisons that differed significantly between treatment groups and had a false discovery ratio (FDR) below 5%. Colored blocks on the right *y*-axis correspond to regional grouping by larger brain areas. Imaging data were analyzed by two-way ANOVA with 5% FDR correction. **C** Graph of brain regions displaying differences by treatment using relative volumetric analysis. Data expressed as percent change with values collapsed between time point and sex. **D** Visual representation of brain regions displaying relative volumetric differences in MAR-ASD offspring compared to control animals in males, females, or both sexes combined. Spinal trigeminal nucleus not pictured due to orientation of rendering. Also, subregions within brain area are not broken down (i.e., sensory dysgranular cortex in males) to reduce complexity. All regions are detailed in Supplementary Table 2.

Despite similar behavioral outcomes between males and females in response to treatment, sex-specific differences in global brain volumes were observed in MAR-ASD offspring. Specifically, while male MAR-ASD rats exhibited a decrease in total brain volume (TBV) compared to sex- and age-matched controls, female MAR-ASD rats instead displayed a volumetric increase in TBV (Fig. 4A). These effects in response to treatment appeared persistent in male offspring, as they were observed at both PND30 and PND70. In contrast, female MAR-ASD offspring displayed significant differences in TBV only at PND70. To understand which regional effects may underlie the differences seen in TBV, we conducted both atlas-based segmentation (ABS) and voxel-wise deformation-based morphometry (DBM) analyses of regional brain volumes. Using absolute volumetric measurement (mm^3^), ABS data revealed that female MAR-ASD rats displayed significant increases across nearly all brain regions examined (94%, 108/115 regions at 5% FDR) suggesting a global effect of increased brain volume in response to treatment. The effects of treatment in male MAR-ASD animals were comparatively more modest, with only a handful of regions passing strict FDR correction (*q* < 0.05) (19%, 22/115 regions) (Fig. 4B and Table S2) Furthermore, the direction of regional effects in MAR-ASD rats corroborated those differences seen in TBV. Given the directionality of treatment-induced effects by sex, with females displaying an increase and males a decrease in absolute total and regional volumes, analyzing both sexes together abolished any statistically significant effects (Fig. 4B; “Both”). To correct for the fact that absolute volumetric findings are influenced by differences in TBV, as well as to understand any treatment-specific effects lost due to MAR-ASD effects by sex, we also evaluated the dataset using relative brain volume (% of TBV) as a metric. Comparing relative regional volumes, we found that the number of significantly different regions passing FDR correction was dramatically reduced in both sexes, particularly in females. Only two regions remained significantly different (5% FDR) in relative regional analysis of MAR-ASD female offspring compared to control females, the periaqueductal gray (PAG) and the substantia nigra (SN) (Fig. 4B). Analysis of MAR-ASD male rats, on the other hand, revealed effects that were restricted to the sensory cortex and the cerebellum, with volumetric increases seen in areas of the sensory cortex alongside decreases in cerebellar volume (Fig. 4B). Additionally, a treatment-specific regional phenotype emerged when evaluating relative volumetric results, in contrast to analysis using absolute volume. Irrespective of sex, MAR-ASD rat offspring exhibited volumetric differences in midbrain and cerebellar regions compared to controls. This included the PAG, inferior colliculus, and pretectum, as well as the inferior cerebellar peduncle and cerebellar gray matter (Fig. 4C, D). To complement the ABS analysis, we also carried out a voxel-wise DBM analysis, the results of which broadly supported the data derived using ABS analysis. Specifically, absolute volumetric analysis using a voxel-wise DBM approach showed that clusters of voxels with significantly increased volumes were apparent across the brains of MAR-ASD female offspring (Fig. S5). The same analysis of male MAR-ASD offspring did not reveal any voxel clusters that were significantly different by treatment using 5% FDR correction. Relative voxel-wise effects by treatment were mostly absent, with exception to data analyzed when both sexes were combined where clusters of voxels with significantly increased volume were seen in the midbrain and dorsal thalamus, while voxel clusters with significantly decreased volumes were observed in the cerebellum and ventral cortical association areas (Fig. S5). Discrepancies between ABS and voxel-wise DBM analysis likely reflect the larger number of comparisons inherent in voxel-wise analysis compared to atlas-based data where changes are averaged across an entire region of interest (ROI).

Interestingly, despite male MAR-ASD rat offspring displaying a decrease in TBV, assessment of relative regional differences (normalized against TBV) revealed a volumetric increase in several regions within the somatosensory cortex (SSC) in male MAR-ASD rat brains. This included the dysgranular zone (S1DZ), a region of interest in other immune-mediated models of ASD (Figs. 4B, D and S6A; [33]). Furthermore, comparing our current findings to sMRI data collected in the MAR-ASD mouse model in adulthood, there appeared to be subtle effects on SSC volume in male MAR-ASD mice [18]. While there were no treatment effects by region in male MAR-ASD mice that passed the stringent FDR cutoff (FDR < 5%), six out of nine regions within the primary SSC were significantly different prior to multiple comparison correction (*p* < 0.05, *q* > 0.05), and these overlapped to a surprising extent with those regions significantly enlarged in male MAR-ASD rats (Fig. S6B). Overall, these findings suggest that MAR-ASD exposure results in changes to offspring brain volume that are cross-species, sex-specific, and persistent over time.

### Altered neurometabolites and relationship with structural findings

**Fig. 5:**
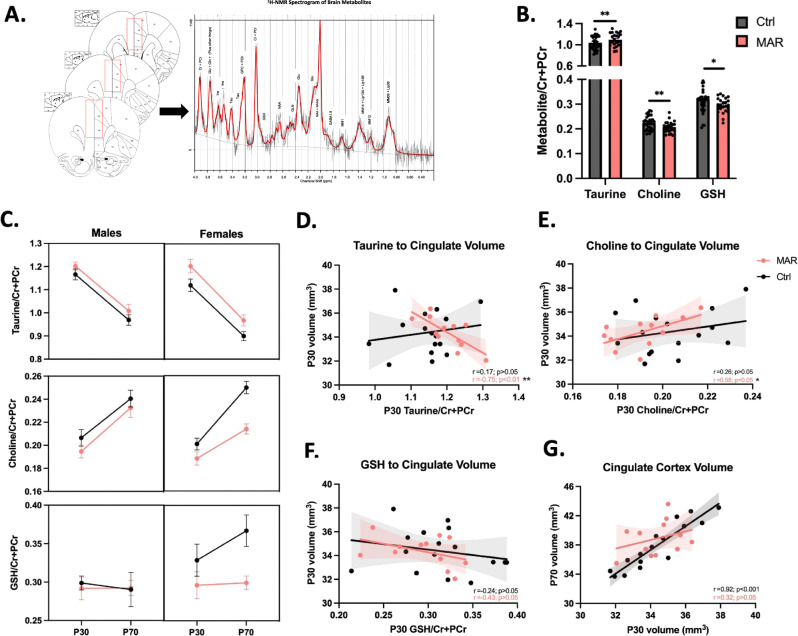
MAR-ASD exposure alters neurometabolite levels in the mPFC. **A** Diagram detailing anatomical location of voxel placement with rat brain atlas as a backdrop. Also depicted is a representative ^1^H-NMR spectrogram output that was used for model fitting and analysis. **B** Metabolites found to differ significantly in response to treatment including taurine, choline, and glutathione (GSH). **C** Metabolite differences split by sex for each metabolite. Metabolite data were collected and analyzed at the time of MR scanning, PND30 and PND70. Data correspond to mean ± SEM analyzed using repeated measures regression analysis, *p < 0.05, **p < 0.01. MAR-ASD; *N* = 6/sex, Ctrl; *N* = 8/sex. Graphs depicting the relationship between metabolite levels and volume of the voxel region at P30 for taurine (**D**), choline (**E**), and GSH (**F**). **G** Correlation between cingulate cortex volume at PND30 and PND70 graphed by treatment. Pearson’s *r* value and related test statistic reported as the result of correlational analysis (**D**–**G**). ^1^H-MRS data were collected across imaging sessions with two timepoints per animal.

While collecting sMRI data, we were also able to interrogate the level of various soluble metabolites present in a predefined recording region using proton (^1^H) magnetic resonance spectroscopy (MRS). This method returns a ^1^H nuclear magnetic resonance (NMR) spectrogram from which metabolite concentrations can be quantified [34]. For this study, the recording voxel was placed in the medial prefrontal cortex (mPFC) (Fig. 5A), due to the involvement of this area in ASD [35] and previous evidence of neocortical abnormalities in response to MAR-ASD aAb exposure [20]. Spectral analysis was performed using LCModel software with a basis set of 26 unique metabolites. Following quality control, we were able to confidently analyze 13 of these metabolites and found treatment-specific differences in offspring exposed to MAR-ASD aAbs (Fig. S7A and Table S3). Specifically, we observed an increase in the level of taurine (*p* < 0.01) in the mPFC (Fig. 5B), accompanied by a decrease in total choline (*p* < 0.01) and glutathione (GSH) (*p* < 0.05) in MAR-ASD offspring compared to controls (Fig. 5B). However, it is important to note that when including full width at half maximum (FWHM) as a covariate the differences in GSH levels were no longer significant, as a positive correlation was observed (Fig. S5C). The best-fit model used for analysis determined that group differences did not vary by age or sex. However, a sex stratified representation of the same dataset revealed that neurometabolite differences in MAR-ASD rat offspring may be driven predominantly by females (Fig. 5C).

Given that sMRI and ^1^H-MRS data were collected in tandem, we were able to evaluate the relationship between metabolites and brain volume in exposed offspring. The region within our brain atlas that aligned the closest with the ^1^H-MRS recording voxel was the cingulate cortex. Therefore, we conducted correlational analysis between the cingulate cortex and those metabolites found to differ significantly by treatment. Interestingly, we observed that taurine displayed a strong negative correlation (*r* = −0.75, *p* < 0.01) with cingulate cortex volume in MAR-ASD offspring at PND30, a finding that was absent in control animals (Fig. 5D). This finding suggests that higher levels of taurine corresponded to lower cingulate cortex volume. Similarly, a relationship was observed between choline and cingulate volume in MAR-ASD animals that was not present in controls. Specifically, choline displayed a positive correlation to cingulate cortex volume in MAR-ASD offspring (*r* = 0.58, *p* < 0.05) (Fig. 5E) suggesting that higher levels of choline relate to greater regional volume. No significant relationships were seen between metabolites and cingulate cortex volume at PND70 or between cingulate cortex volume and the level of glutathione in either MAR-ASD or control offspring (Fig. 5F). Taurine is an organic osmolyte thought to play a role in early inhibitory signaling [36], while choline is a precursor to a range of compounds including acetylcholine, myelin, and cell membrane lipids [37]. Therefore, differences in taurine and choline could potentially contribute to the altered brain development seen in MAR-ASD offspring. As an additional method to examine this relationship, we compared the development of the cingulate cortex between PND30 and PND70 within MAR-ASD and control animals. As expected, control offspring displayed a strong positive correlation (*r* = 0.92; *p* < 0.001) in cingulate cortex volume over time (Fig. 5G), indicating that this region experienced a consistent rate of growth with age. However, offspring exposed to MAR-ASD aAbs did not exhibit the same relationship, as a significant correlation within cingulate cortex volume over time was absent in MAR-ASD animals (Fig. 5G), potentially speaking to dysregulated development of this region in response to treatment.

## Discussion

Accumulating evidence supports the concept of maternal aAbs in ASD [16, 17, 38]. Only recently however, with the creation of robust, translationally relevant model systems [19, 39], are we able to properly investigate the structural and molecular underpinnings of this ASD sub-phenotype. The first goal of this study was to ensure proper construct validity within the MAR-ASD rat model. To this end we confirmed that aAb production in MAR-ASD rat dams is robust, persistent, and does not result in overt inflammation at the time of breeding. The latter aspect is particularly important as several animal models of autism exist that include active inflammation during gestation as a driving factor, also known as maternal immune activation (MIA) models [24, 40, 41]. This concept is methodologically separate from what is seen in the MAR-ASD model where offspring outcomes are influenced instead by the presence of circulating aAbs in dams.

To support the concept of Ab-induced pathology in the MAR-ASD rat model, we confirmed that antigen-specific IgG was able to cross from dam to offspring in appreciable quantities. Moreover, IgG deposition was observed in the brains of MAR-ASD offspring, with specific regional and cell-type distribution. Normally IgG levels in the adult brain are extremely low, with brain IgG concentrations in adult rats observed to be ~500x lower than that seen in blood plasma [42]. However, IgG transferred to offspring during gestation can access the brain prior to closure of the blood-brain-barrier (BBB) [43] and theoretically persist for some time due to the long half-life of IgG (~3wks) [44]. Despite recent work suggesting a role for immunoglobulin signaling in glial development [45], few studies to date have characterized the extent of IgG deposition in the brain during perinatal development. It is important to note that we did not compare the levels of brain IgG between MAR-ASD and control offspring in this study. Additional work is underway to determine the extent of IgG deposition in the brains of exposed offspring, including region-specific distribution patterns, the range of affected cell types, and the extent to which this differs between treatment and control animals across the lifespan.

Since we were able to detect IgG in the brains of MAR-ASD exposed offspring, we sought to evaluate if the levels of aAb target proteins were altered in response to exposure. A difference in the levels of target proteins could be a proxy for aAb impact on protein viability. WB analysis of either frontal brain or cerebellar lysates suggested that the levels of LDH, STIP1, and CRMP1 proteins were not significantly altered in response to MAR-ASD aAb exposure. While protein levels were found to be similar in MAR-ASD and control offspring, it does not necessarily rule out potential impact of MAR-ASD aAbs on target protein function. Previous epitope mapping of MAR-ASD aAb binding sites revealed that the antigenic targets flank key phosphorylation sites important for protein function [46]. Therefore, future studies will include analysis of target protein phosphorylation status as well as enzymatic function where applicable. Ultimately, it will be key to understand whether MAR-ASD aAbs mediate their effects by binding to target proteins in situ or if off-target effects also occur, such as effects at the maternal-fetal interface where impact on placental function may play a role.

As ASD is a behaviorally defined disorder, we examined whether MAR-ASD aAb exposure altered behavioral outcomes in rat offspring. Previous passive transfer models in mice and non-human primates (NHP) revealed changes in behavioral development of offspring following MAR-ASD aAb exposure, including alterations in repetitive behaviors and species-typical social behavior [27, 47]. Furthermore, an advanced antigen-driven model initially created in mice revealed that mouse offspring exposed to endogenously produced MAR-ASD aAbs exhibited a decrease social play behavior, an increase self-grooming behavior, and altered USVs across the lifespan [19]. Here we report that MAR-ASD exposed rat offspring exhibit alterations in several of these behavioral domains, including a reduction in pup USVs and aberrant interactions with an unfamiliar conspecific. While the functional significance of pup USVs has been debated [48], a reduction in USVs may be indicative of early communication deficits and raises the possibility of circuit vulnerability in later social development. In support of this concept, MAR-ASD rats demonstrated a reduction in species-typical social behavior when interacting freely with a novel animal. MAR-ASD rats displayed pronounced group differences in the incidence and duration of social play behavior, which is important to normal social and cognitive development in rats [49]. The absence of social deficits on the three-chamber sociability and social novelty assay suggests that prenatal exposure to MAR-ASD aAbs does not result in a global reduction in social interest, but may disrupt neural systems that support more complex, reciprocal social interactions. Mounting evidence suggests that multimodal measurements of social behavior, which includes analysis of freely interacting animals, may provide a more comprehensive assessment of species-typical alterations in social development [50–52]. Given that increased repetitive behaviors have been reported in our previous MAR-ASD models in mice [19], we also quantified the frequency and duration of self-grooming during social dyads. Although not statistically significant, trend level differences suggest that MAR-ASD rat offspring may engage in self-grooming behavior more frequently, which warrants further investigation. The absence of group differences in elevated plus maze, open field and PPI indicates that MAR-ASD offspring exhibit species-typical levels of anxiety, exploratory locomotion, and sensorimotor gating abilities [53, 54].

To understand what may be driving these alterations in behavior, we conducted longitudinal sMRI analysis of offspring brain volume at P30 and P70. These ages were chosen to represent a pre-pubertal and a post-pubertal time point, respectively. The goal was to explore the potential contribution of sex hormones on the effects seen, as certain brain regions have been shown to exhibit sexually dimorphic volumetric changes following puberty in rodents [55]. However, there were no significant treatment × time interactions on regional volumes in either sex of MAR-ASD rat offspring that passed FDR correction (FDR < 5%), suggesting that either the effects of treatment did not differ by age, or we were unable to detect those differences. Effect size (partial eta squared) analysis of treatment × time interactions would support the latter argument as a few regions displayed large effect sizes at one time point opposed to another, particularly in male rats. For example, in certain subregions, relative volumetric differences appeared more pronounced at either PND30 or PND70 (Fig. S4A, C and Table S1). Analysis of TBV across time in rat offspring, revealed sex-specific effects in that male MAR-ASD rats displayed a decrease in TBV while females displayed an increase in TBV. These results are consistent with our previous findings in mice, where we observed brain-wide increases in total and regional brain volumes in female mice exposed to MAR-ASD aAbs. No differences in brain volume however were observed in male MAR-ASD mice. The discrepancy between these findings may be attributable to species-specific responses to aAb exposure and brain development trajectories between mice and rats [56, 57].

Differences in regional volumes following MAR-ASD aAb exposure also varied depending on sex and the analysis method employed. Specifically, using ABS, nearly every brain region in female rats displayed an absolute volumetric increase, reflecting the increases in TBV in these animals and suggestive of isometric regional scaling (i.e., regions increase linearly with TBV). However, when correcting for TBV the affected regions passing FDR cutoff (<5%) were reduced to two: the PAG and the SN. This may reflect allometric scaling of these regions relative to TBV that could be indicative of abnormal neurodevelopment and/or connectivity [58]. The PAG and SN are both located in the midbrain, with evidence for bidirectional connectivity between these regions [59, 60]. While the SN is known for its role in movement disorders and reward signaling, and the PAG in processing of fear memories, evidence suggests that both regions are important for descending pain perception [61, 62]. Interestingly, altered pain sensitivity is a known phenomenon in ASD [63]. Whether this could be a result of modified descending pain pathways over effects on higher order sensory processing remains to be determined. Further, the PAG is also thought to be important for controlling a broad range of social behaviors, as part of the “social behavior neural network” [64]. This in addition to acting as a gate for ultrasonic vocalization production [65], contextualizes the potential relevance of the PAG in respect to the behavioral differences observed in this study. Similar to females, male MAR-ASD rats showed a substantial reduction in the number of affected brain regions after correcting for total brain volume, again suggesting that most brain regions in these animals were normally scaled to TBV. Differences in relative brain volumes were observed within regions of the cerebellum and sensory cortex in males, with a surprising increase in brain volume seen in regions of the sensory cortex, despite a decrease in TBV in male MAR-ASD rats. Interestingly, recent research has reported substantial connectivity between the cerebellum and sensorimotor/prefrontal areas in mice and rats [66, 67]. Additionally, these cerebellar-cortical circuits have been shown to regulate social and repetitive behaviors [67, 68]. When examining both sexes together a treatment-specific phenotype was apparent in MAR-ASD rat offspring irrespective of sex, defined by volumetric increases in the thalamus and midbrain regions accompanied by a decrease in cerebellar volume. Functional connectivity studies will be necessary to understand whether these changes may reflect altered signaling between these regions.

Analysis of ^1^H-MRS data collected from MAR-ASD and control offspring showed an increase in taurine alongside decreased levels of choline-containing compounds and glutathione in the mPFC of MAR-ASD aAb exposed animals. Taurine is thought to be a gliotransmitter in the brain, enriched in astrocytes, and found to influence a range of neurodevelopmental events, including oligodendrocyte maturation, neural progenitor cell (NPC) proliferation, and synapse development in animal studies [69–71]. Interestingly, both plasma and brain taurine levels have been implicated in ASD [72], and was recently also found to be differentially expressed when the microbiota from autistic individuals was transplanted into germ-free mice [73]. It is important to note that taurine, and all other metabolites measurable using MRS, are indicative of the metabolite in its soluble form as rotational movement is needed within the molecule for proton resonance [34]. This is particularly important when interpreting the reduction in choline seen in MAR-ASD rat offspring. Choline-containing compounds are a major component of the cell membrane. However, the choline signal measurable via MRS is predominately reflective of phosphocholine and glycerophosphocholine involved in active membrane turnover and, to a lesser extent, acetylcholine in the brain [34, 37]. Choline-containing compounds also act as precursors for the synthesis of myelin. Myelination has been shown to be a dynamic process across the lifespan [74], and white matter abnormalities are a common finding in ASD [75]. Therefore, it could be the case that altered choline levels may also reflect effects on myelin turnover. Finally, glutathione (GSH) levels were found to be reduced in MAR-ASD rat offspring compared to control animals. GSH is a potent antioxidant that maintains the redox state in cells and protects against cellular stress [76]. Oxidative stress has long been implicated in ASD, with lower levels of GSH seen in the plasma [77] and brain [78] of individuals with ASD. Of note, no differences were observed in the levels of GABA, glutamate, or in the ratio of these metabolites in response to MAR-ASD exposure.

**Fig. 6:**
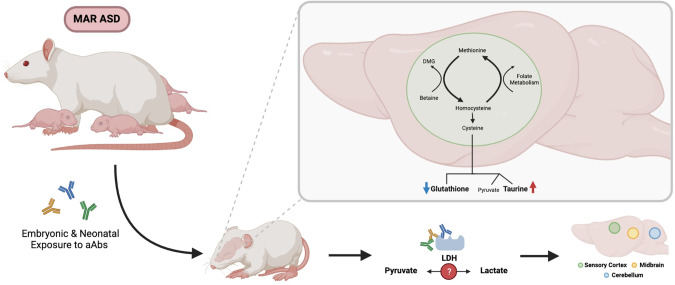
Schematic depicting the effects of MAR-ASD aAb exposure on offspring. Antibodies generated by dams transfer to offspring during gestation and impact molecules involved in the cysteine metabolic pathway. This could be the result of aAb effects on target proteins or those involved in this pathway thereby affecting the homeostatic balance and potentially leading to the noted changes in brain volume.

Interestingly, taurine and GSH fall within the same metabolic pathway: the cysteine:methionine metabolic pathway, both being downstream products of cysteine metabolism (Fig. 6). Cysteine metabolism also results in the production of pyruvate, although pyruvate produced via this mechanism is only a minor contributor to the overall pool of pyruvate within a cell. Of interest, the interconversion of pyruvate to lactate, crucial for glycolysis, is catalyzed by the enzyme LDH. LDHA and LDHB are clinically relevant protein targets for MAR-ASD aAbs and were used as antigens to develop the MAR-ASD rodent model described herein. Moreover, existing literature suggests that taurine can modify the expression of a range of targets involved in cellular stress including LDH, CRMP-family genes, and chaperone molecules such as STUB1 (STIP1 homology and U-box containing protein 1) [79–81]. Extrapolating upon this, it could be that aAb targeting of LDHA, LDHB, CRMP1, STIP1, or homologs thereof, may affect protein function. For example, aAbs to LDH could impact the ability to facilitate the pyruvate:lactate conversion, resulting in a metabolic imbalance and effects on brain development (Fig. 6).

Inherent limitations exist in interpreting the data discussed in this study. Firstly, we hypothesize that MAR-ASD aAbs mediate their effects through binding to protein targets and affect protein expression or function. We did not however find evidence of altered MAR-ASD protein expression levels using WB analysis of brain tissue. Of note, this does not discount the possibility that aAbs could alter protein phosphorylation status and protein function directly, an area of active examination. Additionally, we have yet to complete a thorough characterization of brain aAb deposition by treatment. It could be that MAR-ASD pathology is actually a result of indirect mechanisms rather than direct effects on the brain, such as through alterations in placental signaling which have been linked to disrupted brain development and behavior in other studies [82]. Finally, due to behavioral testing across the lifespan and the nature of longitudinal imaging, separate cohorts were used for sMRI/S and behavior in the current study. Therefore, we are unable to make any direct sMRI/S-behavioral associations and can only theorize on the relationship between regional volumetric differences and behavioral outcomes. Since we only observed behavioral differences in a handful of tests, future studies can be more refined in scope allowing greater insight in brain/behavior relationships and expansion of sample sizes to detect more subtle treatment effects.

In conclusion, this study reveals that gestational exposure of rats to MAR-ASD aAbs results in longitudinal, sex-specific effects on offspring outcomes including behavior, brain structure, and neurometabolism. Strengths of this study include the translational nature of the methods used, particularly regarding sMRI and ^1^H-MRS, for which there exists a clinical literature base with respect to ASD. Future work will be focused on understanding the molecular mechanisms underlying MAR-ASD neuropathology.

### Supplementary information

Supplemental methods and results

Supplementary Table 1

Supplementary Table 2

Supplementary Table 3

## Data Availability

Data that is not already available in the main text or Supplementary Materials are available from author upon request.
