# Supplementary material for: Altered behavior, brain structure, and neurometabolites in a rat model of autism-specific maternal autoantibody exposure
Source: Mol Psychiatry. 2023 Mar 27;28(5):2136–47. doi: 10.1038/s41380-023-02020-3 (PMC10575787; doi:10.1038/s41380-023-02020-3)
Supplement: Supplementary file 1 — Supplemental methods and results [file 41380_2023_2020_MOESM1_ESM.docx]

**Supplemental Methods, Results and Figures**

**Title: Altered behavior, brain structure, and neurometabolites in a rat model of autism-specific maternal autoantibody exposure**

**Running title:** MAR-ASD and behavior, brain, and neurometabolites

**Authors:** Matthew R. Bruce^1^, Amalie C.M. Couch^2,7^, Simone Grant^3^, Janna McLellan^1^, Katherine Ku^3^, Christina Chang^3^, Angelica Bachman^3^, Matthew Matson^3^, Robert F. Berman^4,9^, Richard J. Maddock^3^, Douglas Rowland^5^, Eugene Kim^6^, Matthew D. Ponzini^7^, Danielle Harvey^7^, Sandra L. Taylor^7^, Anthony C. Vernon^2,8*^, Melissa D. Bauman^3,9*^, Judy Van de Water^1,9*^

**Affiliations:**

^1^Department of Internal Medicine, Division of Rheumatology, Allergy, and Clinical Immunology, University of California; Davis, CA, USA

^2^Department of Basic and Clinical Neuroscience, Institute of Psychiatry, Psychology and Neuroscience, King’s College London; London, UK

^3^Department of Psychiatry and Behavioral Sciences, University of California; Davis, CA, USA

^4^Department of Neurological Surgery, University of California; Davis, CA, USA

^5^Center for Molecular and Genomic Imaging, University of California; Davis, CA, USA

^6^Department of Neuroimaging, Institute of Psychiatry, Psychology and Neuroscience, King’s College, London, UK

^7^Department of Public Health Sciences, University of California; Davis, CA, USA

^8^MRC Centre for Neurodevelopmental Disorders, King’s College London; London UK

^9^MIND Institute, University of California; Davis, CA, USA

^*^Co-corresponding authors.

Communicating author email: [javandewater@ucdavis.edu](mailto:javandewater@ucdavis.edu)

**File Contains:**

Supplemental Methods

Supplemental Results

Figure S1- MAR-ASD antibody levels in offspring and effects on target proteins

Figure S2 - Pre-weaning behavioral outcomes

Figure S3 - Social Dyads, Three-Chamber Sociability and Social Novelty Test.

Figure S4 - Elevated Plus Maze, Open Field and PPI

Figure S5 - Voxel-wise analysis of brain volume

Figure S6 - Relative regional volumetric differences by time, sex, and treatment

Figure S7. Additional MRS metabolite data

**Additional Supplementary Methods**

**Animals**

Animals were kept in standard housing consisting of polypropylene cages (30.5 cm x 35.6 cm x 20.3 cm) with high-top wire lids, cob bedding, and nesting. Red enrichment boxes were provided within cages for dams to enhance quality of life and breeding success (Fisher Scientific; Cat# 14-726-564). Breeding was conducted 2 weeks following the final treatment (described below) to produce two separate cohorts for the behavioral and neuroimaging studies. Each cohort consisted of 5-6 dams per treatment group for behavior and 3-4 dams per treatment group for neuroimaging. After birth, animals were tattooed for identification purposes and litters culled to 8 (4 male/4 female). To identify rats, pups were labeled by paw tattoo on postnatal day (PND) 2-3 using non-toxic animal tattoo ink (Ketchum Manufacturing Inc., Brockville, ON, Canada) and later tail-marked with permanent marker at weaning (PND21) to allow investigators to run and score behaviors blind to treatment condition. All testing was conducted during the light phase of the 12- hour light/dark cycle.

**Autoantibody Confirmation**

Briefly, Nunco Maxisorp plates (Thermo Scientific, Waltham, MA) were incubated with synthetic proteins (Expression Systems, Davis, CA) corresponding to the 4-core MAR ASD proteins (LDH-A, LDH-B, STIP1, CRMP1), at a concentration of 2ug/mL. Plates were incubated overnight at 4dC, washed with PBST the next day, blocked with 1% Protein Free Blocking Buffer (Thermo Scientific, Cat# 37572), then incubated with rat dam serum samples at a concentration of 1:250 for 30 minutes at room temperature. Plates were then washed with PBST and incubated with a highly cross-adsorbed goat anti-rat secondary antibody (InVitrogen, Cat# 62-9520) for another hour. Following additional washes in 1X PBS to remove detergent, plates were incubated with TMB substrate (3,3′, 5,5′-tetramethylbenzidine; BD OptEIA, San Jose, CA) and neutralized using 2N H_2_SO4 to result in colorimetric substrate deposition. Data were read on a microplate reader at an optical density of 450 nm.

**Longitudinal Behavioral Testing**

Pre-weaning behavioral assays were performed on PND 4, 8, and 12. These assays included ultrasonic vocalizations for early communication and early developmental milestones, which were scored on an ethogram described below. On PND 26 and 96, anxiety-like behaviors were assessed in subject animals on an elevated plus maze. On PND 29 and 100, subject rats had exploratory locomotion assessed in an open field maze, to control for potentially confounding effects of sedation or hyperactivity on the sociability assays. Social approach in an automated three-chambered apparatus was used to assess sociability in subject animals at PND 27-28 and 97-98. This task used methodology similar to protocols described previously for mice (*1*) and rats (*2*). On PND 34 and 102, rats were tested in pre-pulse inhibition (PPI), a task used to assess sensorimotor gating in rats. Reciprocal social dyad interactions were evaluated at three developmental timepoints: juvenile (PND 36-37), young adult (PND 55-56), and adult (PND 103-104). Untreated stimulus partners were matched in age, strain, and sex and housed in the same vivarium, but were unfamiliar to test rats. The amount of time spent in nonsocial activity, self-grooming, social interaction, social proximity, and social play were quantified using Noldus Observer software (Noldus Information Technology, Netherlands). Before each trial, all testing chambers were thoroughly cleaned and disinfected with 10% Nolvasan solution (Fort Dodge Animal Health, Fort Dodge, IA). For pre-weaning behavioral experiments, the litter (6 MAR-ASD dams (N= 23M/25F); 5 control dams (N=20M/20F)) was temporarily removed from the dam on PND 4, 8, and 12 to evaluate developmental milestones and to quantify isolation-induced pup ultrasonic vocalizations (USVs). For post-weaning behavioral experiments, 2 male and 2 female offspring from each dam (6 MAR-ASD dams (N= 12M/12F); 5 control dams (N=10M/10F)) were selected at random following weaning at PND 21 to participate in the behavioral test battery.

Developmental milestones**:** On PNDs 4, 8, and 12, subject animals were removed from the dam for developmental milestones, including body temperature, body weight, cliff avoidance, fur development, head width, incisor eruption, negative geotaxis, pinnae detachment, righting reflex, and tail length. Fur development, incisor eruption, and pinnae detachment were given a score between 0-3 depending on the pup’s development. Cliff avoidance was tested by placing the pup at the edge of a precipice with their paws just over the edge. A score between 0-3 was given based on the degree of the response. To measure negative geotaxis, rat pups were placed facing downward on a wire mesh slanted at a 45-degree angle. Pups were given a score between 0-3, depending on their ability to turn face up and climb up the ramp. Righting reflex was tested by placing the pup on its back and recording the latency to reach an upright position with all four paws flat on the surface. This was done twice and the average of the two latencies was taken as the overall righting reflex value.

Isolation-induced pup 40-kHz ultrasonic vocalizations: On PNDs 4, 8, and 12, subject pups were removed individually from the nest at random and gently placed into an isolation container (8 cm x 6 cm x 5 cm; open top) made of plastic. The isolation container was filled with a thin layer of clean corncob bedding before the first test session and between each new test subject. The isolation container was placed in a sound attenuating chamber (18 cm x 18 cm x 18 cm) made of 4 cm thick noise-dampening Styrofoam padded walls. An ultrasonic microphone (Avisoft Bioacoustics, Berlin, Germany) was attached to the chamber roof and hung 7 cm above the chamber floor. Calls were collected for 3 min at a sampling rate of 250,000Hz via the microphone connected to an Avisoft UltraSoundGate 116 USB audio device, which was connected to a computer with Avisoft Recorder software. Immediately following the 3-min recording session, body temperature was taken with a digital thermometer placed on the abdominal surface (TH-5 Thermalert Monitoring Thermometer, Physitemp Instruments, Inc., Clifton, NJ). Ultrasonic vocalization spectrograms were displayed using the Avisoft SASLab Pro software. Pup calls in the ultrasonic range with peak frequencies higher than 20 kHz were identified manually by a trained investigator blind to treatment group.

Elevated plus maze (EPM): On PNDs 26 and 96, subject animals were tested for anxiety-like behaviors in an elevated plus maze task. The maze is a black, polypropylene plus-shaped platform consisting of two opposite enclosed arms (10cm x 50cm) and two opposite open arms (10cm x 50cm). The arms meet at a center square platform (10cm x 10cm), and the enclosed arms are surrounded by 10cm high walls. The entire maze is elevated 100cm off the ground. At the start of each trial, the subject rat was placed on the open center platform facing an open arm. The rat was allowed to explore the apparatus for 5 minutes and was video-recorded using video-tracking software (Ethovision v.4.0, Noldus Information Technology, Netherlands). The tracking software used the midpoint of the body of the rat to distinguish when the test subject entered or exited an arm boundary. Trials were scored on the amount of time spent in the open arm and the number of entries into the open arm.

Open field maze: On PNDs 29 and 100, subject rats were tested for spontaneous locomotor activity. Locomotion was measured using a fully automated contrast-sensitive video-tracking program (Integra Accuscan, Columbus, OH, USA). The setup allowed the simultaneous tracking of four animals, using four separate square observation arenas (41.3cm w x 29.2cm h x 41.3cm l). At the beginning of the trial, the subject animal was placed in the center of the arena. Spontaneous activity was measured over a 60-minute period. Distance moved (cm) was calculated every 1 minute. The sampling rate was set to five samples per second. Parameters used to measure the subjects’ locomotion were distance travelled, time spent in center of arena, horizontal activity, and vertical activity.

Three-chamber sociability and social novelty test: On PNDs 27-28 and 97-98, subject rats performed a social approach and novelty task. Stimulus animals were matched in age, strain, and sex, and were housed in the same vivarium, but were unfamiliar to test rats. Subject and stimulus rats were placed in a dimly lit room (30 lux) and allowed to acclimate to the test room for 5 minutes. The subject rat was then placed in a square, three-chambered box made of clear plastic (101.6cm l x 101.6cm w x 33.7cm h) (Stoelting Co., Wood Dale, IL). The subject rat habituated to the empty three-chambered box for 10 minutes, with free access to all three chambers. Two plastic cylindrical cages (13.3cm diameter x 21.0cm h) were then placed in the left and right chambers. In the first trial, social approach, the first stimulus rat was placed under one of the cages in a side chamber, while the other cage was left empty and placed in the opposite side chamber, serving as a novel object. The subject rat was allowed to explore the entire arena for ten minutes. Stimulus rat and novel object placement alternated between the left- and right-side chambers for each individual subject. In the second trial, social novelty, the subject rat was placed back in the center chamber. The chamber with an empty cage was then replaced with a cage containing a novel stimulus animal, while the previous, familiar stimulus animal remained on the other side of the arena. The subject animal was once again allowed to explore the entire arena for ten minutes. Automated video tracking with EthoVision 10.0 XT (Noldus, Wageningen, The Netherlands) software was used to detect head-directed movement and sniffing behavior during all test phases, as well as to quantify time spent in each chamber, cumulative duration of nose within zone around cup, and the number of transitions between chambers. Typical sociability in the approach phase was defined as spending more time in the chamber containing the stimulus rat than the chamber containing the novel object, and more time spent near the cup with the stimulus rat than the novel object. Novelty preference in the novelty phase is defined as spending more time in the chamber containing the novel rat, and more time spent around the cup with the novel stimulus rat than the original stimulus rat.

Pre-pulse inhibition: On PNDs 34 and 102, rats were tested in pre-pulse inhibition (PPI), a task used to assess sensorimotor gating in rats. The subject rats were placed in a clear cylinder, which was then attached to a piezoelectric transducer platform. The platform was placed in a sound-attenuating chamber outfitted with speakers, which were controlled by specialized software (SR-Labs, San Diego Instruments, San Diego, CA). Subject rats were acclimated to the chamber for 5 minutes with a 65 dB background white noise. They were then presented with a pseudorandom set of 5 different trial types: 120dB startle, 120dB startle with a 74dB pre-pulse, 120dB startle with a 82dB pre-pulse, 120dB startle with a 90dB pre-pulse, and a no stimulus trial, or 65dB white noise. The trials occurred over a 10-minute period. Pre-pulses were introduced 120ms prior to the startle stimuli. Intertrial intervals were randomized between 10 ms and 20 ms. The following equation was used to calculate PPI percentage: PPI = [100 – (pre-pulse/max startle)] x 100].

Social Dyads: Social dyad trials were conducted at three developmental timepoints: juvenile (PND 36-37), young adult (PND 55-56), and adult (PND 103-104). Stimulus partners were matched in age, strain, and sex and housed in the same vivarium, but were unfamiliar to test rats. The social dyad apparatus consisted of three identical Plexiglas chambers (41.9cm w x 29.2 cm h x 41.9 cm l), two side chambers used for acclimation and a center arena to record dyad interactions between the subject and stimulus rat. Both subject and stimulus rats were placed into respective transfer cages and isolated in a quiet, dimly lit (12 lux) room adjacent to the test room for ten minutes, separated by a visual barrier. The subject rat and stimulus rat were then moved to a dimly lit (12 lux) test room and allowed to acclimate for 5 minutes on opposing side chambers. Both rats were placed in the center arena and video recorded using a Sony HDRCX240/B video camera with 2.7-Inch LCD, fixed to a tripod. The rats were allowed to interact undisturbed for 10 minutes. Videos were scored using Observer XT12 software (Observer Version XT12, Noldus Information Technology, the Netherlands). Focal observations were used to quantify the amount of time the subject rat spent in nonsocial activity (not interacting with the stimulus animal) and the amount of time spent in social interactions, further divided into three broad categories: (i): social play – a composite of well-characterized play behaviors including pouncing/playful nape attack, pinning, wrestling, boxing, tail pulling, and chasing (running pace); (ii): social exploration – a composite of social investigation such as sniffing and following (walking pace) or non-play contact such as grooming, licking, crawling over or under; and (iii) social proximity – scored when the rats were within 2cm of each other, but not actively engaged in investigation, contact, or play behaviors. The amount of time spent self-grooming was also quantified.

**Statistical Analysis of Behavioral Outcomes**

Numeric variables were summarized as means ± SD and categorical variables as counts and percentages. Statistical analyses were conducted using R version 4.0.5 (R Core Team 2021; (*3, 4*)).

Developmental Milestones: Developmental milestones evaluated were body temperature and weight, tail length, head width, pinnae detachment, eye opening, incisor eruption, fur development, righting reflex, cliff avoidance, and negative geotaxis. These milestones were evaluated at 3 times points: post-natal day 4, 8 and 12. Because there was no variation in incisor eruption scores at post-natal day 4 and fur development scores at post-natal day 8, these time points were excluded from their respective analysis. Eye opening scores were the same for all groups, sexes, and ages and thus not analyzed. Linear mixed effects models (LMMs) were used to model each milestone as a function of treatment group (MAR or Cntl), sex, post-natal days (4, 8, or 12 days), and all two and three-way interactions. Post-natal days was modeled as a categorical factor. A random subject effect was included for each pup to account for within-subject correlation. The need for a random dam effect to account for correlation among pups from the same dam was assessed using a likelihood ratio test (*4*). If inclusion of a dam random effect was not found to significantly improve model fit, it was dropped and the model refit with only a random intercept for each pup. Residual plots were used to assess model assumptions. A dam random effect was included in all models except for cliff avoidance. Righting reflex was log transformed to meet model assumptions. Non-significant (p > 0.05) interactions were dropped and final models were fit consisting of all main effects but only statistically significant interactions.

Ultrasonic Vocalizations: Isolation-induced USVs are often used to characterize early social communication as pups emit these 40kHz calls following maternal separation to communicate with the dam, facilitating pup retrieval. The number of ultrasonic vocalizations (USVs) were evaluated using a LMM modeling USV as a function of sex, treatment group (MAR or Cntl), post-natal days (4, 8, or 12 days), and all two and three-way interactions. Post-natal days was modeled as a categorical factor. A random subject effect was included for each pup and the need for a random dam effect assessed. A dam effect was deemed necessary based on the results of this test. Residual plots were used to assess model assumptions. A final model containing all main effects, but only statistically significant interactions was fit.

Social Dyads: Social behavior, nonsocial behavior, proximity, and investigation were measured as time spent engaged in each activity. These behaviors were evaluated using a LMM. Each behavior was modeled as a function of sex, treatment group (MAR or Cntl), age (juvenile, young adult, and adult), and all two and three-way interactions. A random subject effect was included and the need for a random dam effect was assessed. Residual plots were used to assess model assumptions. The final model contained all main effects but only statistically significant interactions. Self-grooming and play also were reported as time spent in these activities. However, some rats did not exhibit these behaviors resulting in highly skewed distributions with a large number of 0 entries. To capture both aspects of these data, self-grooming and play were analyzed using two-part mixed effects models. Two-part models consist of two-parts: one that evaluates the percentage of subjects engaging in a behavior and a second part that models the duration of the behavior among those who did exhibit the behavior (*5*). Two-part models test a joint null hypothesis of no difference in the percentage of subjects engaging in the behavior nor in the duration of the behavior among those who did. Rejection of the null hypothesis indicates a difference in the percentage exhibiting the behavior and/or the duration of the behavior; summary statistics are used to assess which parts contribute to an overall significant two-part test. For these models, a mixed effect logistic regression was used to model the occurrence of the behavior and a linear mixed effect regression used for the duration of the activity. Due to sample size concerns, only main effects of treatment, sex and age were considered. A random subject effect was included in each model to account for within-subject correlation over time. Models were fit via maximum likelihood estimation and likelihood ratio tests used to test each main effect separately. The need for a dam random effect was tested but not found to be necessary for any of these models.

Three-chamber sociability and social novelty test: Social approach and novelty experiments consist of quantifying interactions between a novel rat and a novel object (sociability phase) or between a novel rat and a familiar rat (novelty phase). Outcomes are reported as time spent in a chamber or looking at the novel rat/object as well as the number of entries into the chamber with the novel rat/object. Linear mixed effect models were used to evaluate duration metrics (time spent in chamber, time spent looking) and a Poisson mixed effect model was used for counts of the number of entries. Each outcome was modeled as a function of chamber (rat vs. object), group (MAR vs. Ctrl), sex and all two and three-way interactions. For the Poisson models, the log transformed total number of chamber entries was included as an offset. A random subject effect was included and the need for a random dam effect was assessed; inclusion of a random dam was not indicated for any outcome. Final models containing all main effects but only statistically significant interactions were fit. Residuals were examined to assess model assumptions. Juveniles and adults were modeled separately.

Pre-pulse Inhibition: Linear mixed effects models were fit to assess the relationship between PPI and the three different pre-pulse intensities (74dB, 82dB, and 90dB), group (MAR vs. Ctrl), sex and all two and three-way interactions. A random subject effect for each rat was included and the need for a random dam effect was also evaluated; a random dam effect was not indicated. Final models containing all main effects but only statistically significant interactions were fit. Model assumptions were assessed through residual plots. Juveniles and adults were analyzed separately.

Elevated Plus Maze: Outcomes of the elevated plus maze task are time spent in the open arms and the number of entries to the open arms. We first fit a LMM to relate time spent in the open arm to group, sex and their interaction. The need for a random litter effect to account for within-litter correlation was tested and was not found to be needed for either juveniles or adults. Therefore, multiple linear regression models were used. Residual plots were used to assess model assumptions. For final models, we retained all main effects and statistically significant interactions. Juveniles and adults were evaluated separately.

The number of entries into the open arm of the maze was similarly modeled using a generalized linear mixed effects model assuming a Poisson error distribution and a log link. The log transformed total number of entries was included as an offset, thereby modeling the rate of entries. As done for the analysis of time spent in the open arm, we tested the need for a random litter effect. Because a random litter effect was not found to be necessary, a Poisson general linear model was used. Juveniles and adults were modeled separately.

Open Field: In the open field experiment, a rat is placed in an arena alone and allowed to explore for 60 minutes. For each 5 minute increment, the distance the rat travels horizontally, vertically, and total distance are recorded. The time spent in the center of the arena is recorded. Linear mixed effect models were used to model the trajectories of distance traveled over time. We modeled distance or center time as a function of time since the trial started, group, sex and all two and three-way interactions. A random intercept was included for each rat. We tested the need for a litter random effect; model fit was not significantly improved with the addition of a litter effect for any outcome. The midpoint of each 5 minute increment was as used for the time variable and was modeled as a numeric value. All outcomes were square root transformed to meet model assumptions and an autoregressive correlation structure assumed for correlation over time. Final models consisting of all main effects and any statistically significant interactions were fit. Juveniles and adults were evaluated separately.

**Magnetic Resonance Imaging and Spectroscopy**

MR acquisition: MR images were acquired on a small animal horizontal bore 7 T system (Bruker; Karlsruhe, Germany) running ParaVision 5.1 software. Animals were anesthetized using 1.5% isoflurane vaporized in oxygen, administered continuously at 1 liter/minute for the duration of the scanning. PhysMonitor software was used to track body temperature and respiration rate. A remote heating device was used to maintain the body temperature of animals at a set point of 37°C. A 72 mm volume coil was used for excitation and a 4-channel phased array surface coil for signal detection (Bruker). Anatomical T2-weighted scans were acquired using a RARE sequence (Rapid Acquisition with Relaxation Enhancement) (*6*) in axial orientation: RARE factor 8, TR = 6100 ms, TEeffective = 60 ms, matrix size 280 × 200, FOV 35 × 25 mm2 (in-plane resolution 125µm) with 44 continguous 0.5mm-thick slices. For MR spectroscopy, a single voxel of 3.8 x 2.2 x 2.0 mm3 was centered in the frontal cortex. To optimize field homogeneity, after 1st order shims were optimized, shims were readjusted including 2nd order shims. A field map was measured and shims were optimized for the localized spectroscopy using the MAPShim algorithm provided by Bruker in PV5.1. In vivo 1H-MR spectroscopy (MRS) data were acquired with a Point-Resolved Spectroscopy (PRESS) (*7*) with a total acquisition time of 8.5 min per scan (TR/TE = 2500/14 ms, 200 averages, 2048 acquisition data points, spectral width 4006.41 Hz) with VAPOR (variable power and optimized relaxation delays) water suppression (bandwidth 200 Hz) (*8*). A reference scan without water suppression was acquired for frequency and eddy current correction during each acquisition cycle. A total of 3 PRESS scans were acquired one after another.

MR image processing: MR images were visually inspected in native space for artifacts, with no images excluded on this basis. Raw MR images were converted from the manufacturer’s proprietary format to the NIFTI format and processed using a combination of FSL 5.0.10 (*9*), ANTs 2.1.0 (*10*) and Quantitative Imaging Tools 2.0.2 (QUIT, (*11*). First, N4 bias field correction was applied (*12*). Separate P30 and P70 template images were constructed from all bias-corrected P30 and P70 images using the *antsMultivariateTemplateConstruction2.sh* script with three iterations (*13*). Each image was then non-linearly registered to its age-matched study template via sequential rigid-body, affine, and SyN registrations using the *antsRegistration* function. The P30 template was similarly registered to the P70 template, enabling normalization of all images across subjects and time points to the P70 template space. Jacobian determinant maps of the composite deformation fields from the P70 template to each subject were calculated using the *CreateJacobianDeterminantImage* function (ANTs). The P70 template was skull-stripped using the RATS algorithm implemented in QUIT using the *qimask* function (*14*). To enable atlas-based segmentation (ABS) analysis of regional brain volumes, the skull-stripped P70 template was then registered to the publicly available Tohoku rat brain MRI atlas. Because the Tohoku atlas includes only cortical parcellations, the Waxholm Space (WHS) rat brain atlas, which has only subcortical parcellations, was registered to the Tohoku atlas, and the two atlases were modified and merged to obtain a full-brain parcellation consisting of 115 regions of interest (ROI). The Jacobian determinants in each atlas ROI were summed to calculate ROI volumes for each subject. The Jacobian determinant maps were log-transformed to allow voxel-wise estimation of apparent volume changes via deformation-based morphometry (DBM) (*15*).

MR image analysis: The registration quality for each individual subject’s MR images was checked visually and no animals were excluded on this basis. We then automatically extracted volumes for the 115 ROIs comprising the hybrid MRI atlas (see MR image processing). Total brain volume was calculated from the summation of each individual atlas ROI volumes (*16*). Group-level differences in total brain volume (mm^3^) were assessed using parametric 2-way analysis of variance (ANOVA) for each sex separately, with “Time” (P30-P70) as within-subject factor and “Treatment” (MAR/CON) as between-subject factor. These analyses were performed using Prism software (v8.4.2; GraphPad, La Jolla, CA, USA) with α = 0.05. Identical statistics were performed to compare regional volume differences for the absolute (mm^3^) and relative (% total brain volume) volumes of all 115 brain regions of interest (ROI) in the hybrid MRI atlas, with α = 0.05 using R-project (v4.0; R Core Team, 2020). The resulting p(ANOVA) values for each model term (main effects and interactions) were then corrected for multiple comparisons to account for Type I errors across the 115 individual ROIs using the false-discovery rate (FDR) in Prism software (v8.4.2; GraphPad, La Jolla, CA, USA). A threshold of 5% FDR (q<0.05) was considered statistically significant. To calculate the magnitude and direction of volume change for each region between groups, we calculated effect sizes, using Cohen’s F, derived from the partial eta^2^ effect sizes from the ANOVA models for main effects of treatment, time or treatment*time interaction. The regional brain volume analysis was run for both sexes combined and for male and female rats separately. For DBM, the voxel-wise analysis of group-level differences in absolute volumes was carried out on the log-transformed Jacobian determinant maps using the Multivariate and Repeated Measures (MRM) MATLAB toolbox. As with the ABS analysis, parametric 2-way ANOVA was run for both sexes combined and separately, using Wilks’ λ as the test statistic, voxel-level thresholding, permutation testing (5000 permutations), and FDR correction (5%, q<0.05).

^1^H-MRS Analysis**:** PRESS data were acquired over 3 separate subscans of 200 averages each, analyzed using LCMODEL, and averaged together after phase and frequency correction for estimation of metabolite concentrations. An analysis window between 0.2 to 4.0ppm was chosen with phase and frequency alignment using LCMODEL. Data were fitted in LCMODEL using a simulated basis set with 26 metabolites, including macromolecule and lipid resonances. Metabolites with Cramér-Rao lower bounds <15% were included in final analysis. To adjust for unknown scaling factors in ^1^H-MRS acquisition, metabolite data in this study were normalized against total creatine levels (creatine + phosphocreatine (Cr+PCr)). Ratio normalization of signal intensity using creatine is widely used in ^1^H-MRS due to the strong signal of creatine in the spectral output and low variability across brain areas and experiments (*17*). To ensure that there were no alterations in creatine levels due to treatment, which might affect results, we also conducted a water-scaled analysis to evaluate raw creatine levels between treatment groups. No significant differences were seen in creatine (Cr), phosphocreatine (PCr), or Cr+PCr between MAR-ASD and control offspring at either time point examined (fig. S5B). Additionally, the results of water-scaled analysis on other metabolites did not differ from creatine-normalized results (Table S3). Eight metabolites were identified as primary outcomes of interest (Gln, Glu, GPC+PCh, GSH, Ins, MM09+Lip09, NAA, tau), while an additional five (Asp, GABA, Glu+Gln, MM14+Lip13a+L, NAA+NAAG) were identified as secondary outcomes. Factors of interest included treatment group (Control, Treatment), sex, and age (30, 70 days). Main effects and interactions among these variables were considered. All animals had observations at both 30 and 70 days. Repeated measures regression, assuming a compound symmetric covariance matrix, was used to assess differences by treatment, sex or age. Model building for each metabolite was similar, using Akaike Information Criterion to identify the best model. Due to the small number of animals, robust standard errors were used. Secondary analyses included a data quality metric (full width-half max (FWHM)) as a covariate. Results were generally consistent in these secondary analyses, unless otherwise stated.

**Additional Supplementary Results**

**Autoantibody Confirmation and Protein Analysis**

**
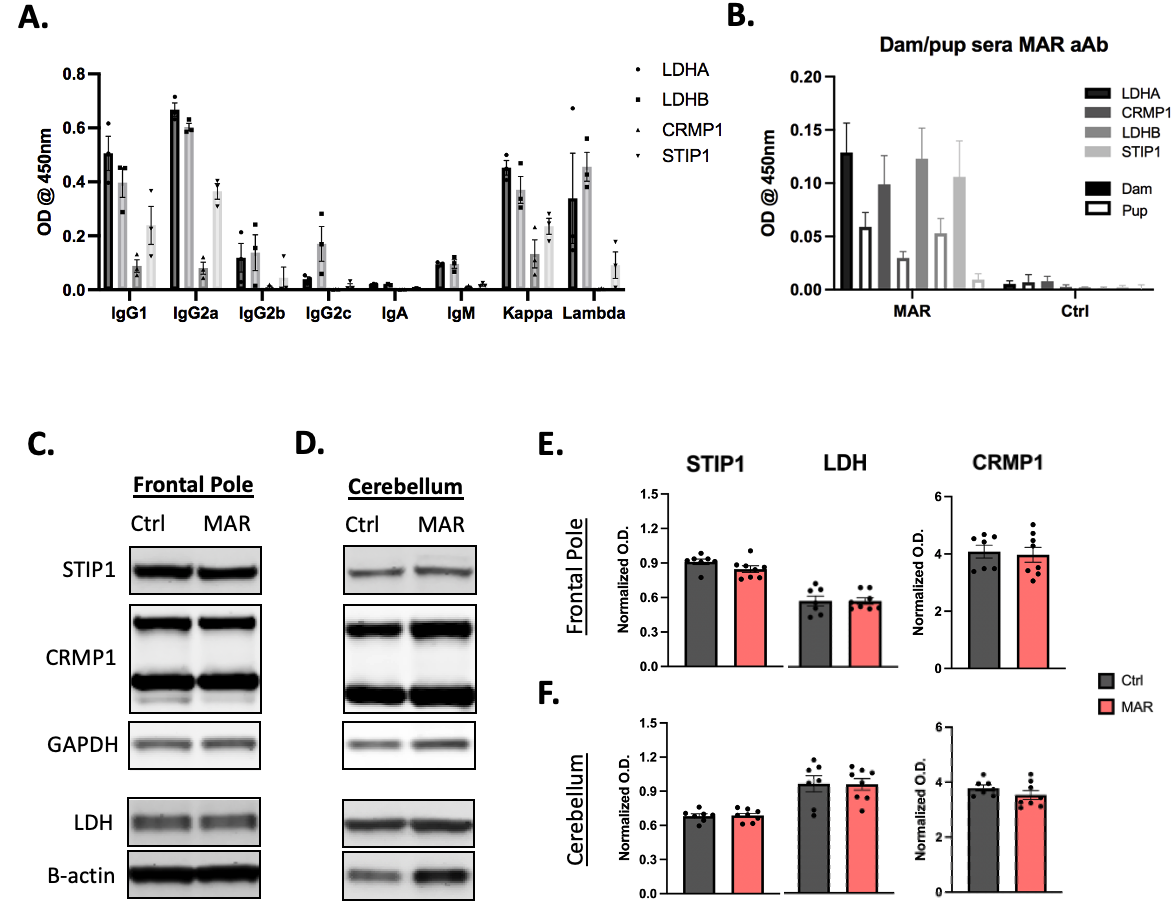
**

**Figure S1. MAR-ASD antibody levels in offspring and effects on target proteins. (A)** Distribution of antigen-specific antibody isotype and subclass in MAR-ASD offspring at PND2. Data expressed as optical density (OD) values from a custom ELISA assay recorded at 450nm. **(B)** Raw OD values for dam/pup total antigen-specific IgG levels. Data split by treatment, solid-colored bars represent dam values with clear bars being offspring at PND2. **(C & D)** Representative western blot reactivity for MAR-ASD protein targets with appropriate loading controls from either the Frontal Pole **(C)** or the Cerebellum **(D)**. **(E & F)** Quantification of western blot results for each brain area. MAR ASD; N=8, Ctrl; N=7, data expressed as mean +/- SEM.

**Behavioral Results**


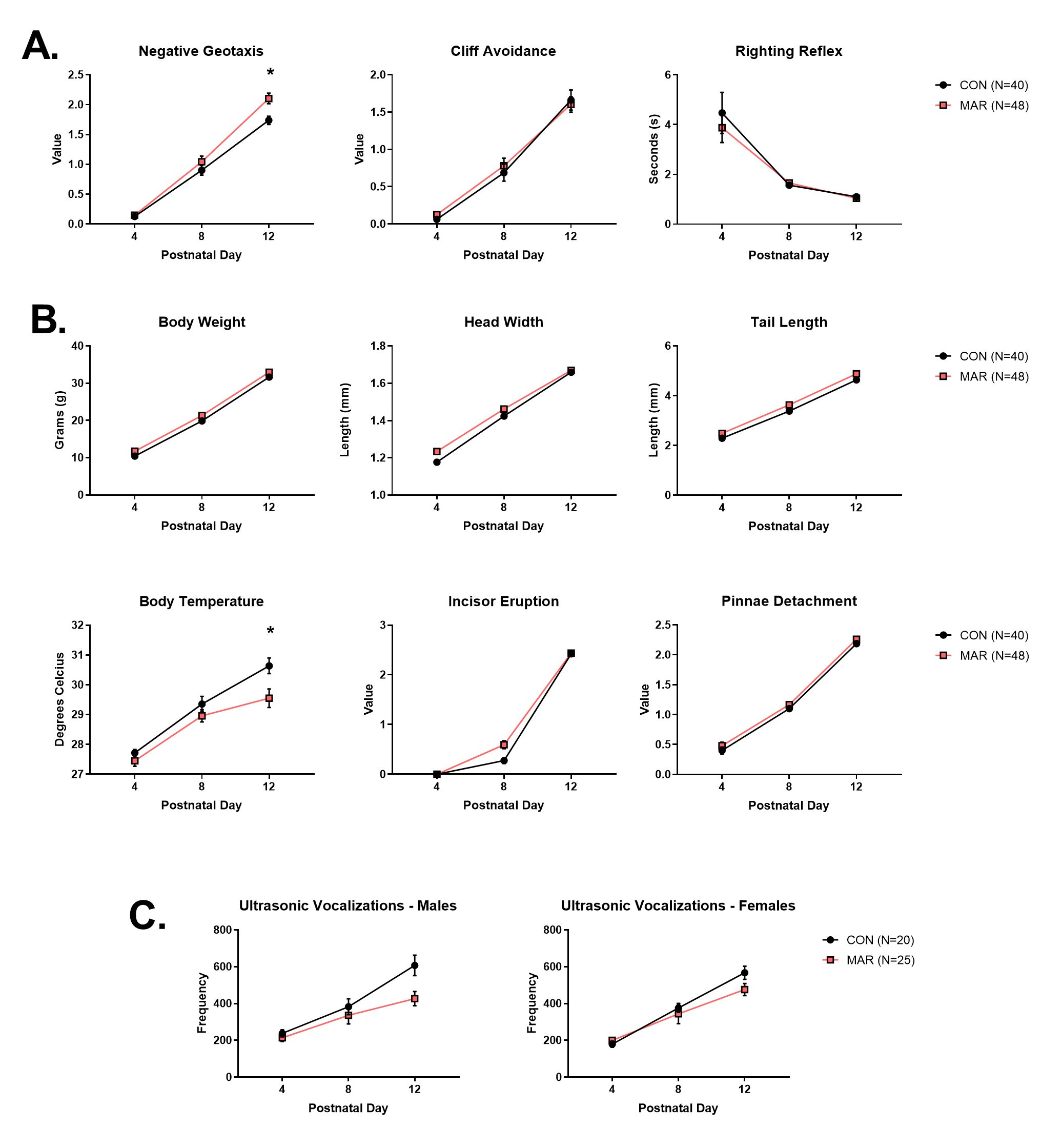


**Figure S2. Pre-weaning behavioral outcomes.** Data showing pre-weaning developmental milestone reflexes **(A)** and physical outcome measures **(B)**. Analysis of developmental milestones in these animals revealed that MAR-ASD rat offspring also had a significantly lower average body temperature (p=0.017; fig. S2B) and increased negative geotaxis (p=0.014; fig. S2B) at postnatal day 12, suggesting a potential impact of MAR-ASD exposure on directional reorienting behavior, but not in cliff avoidance or righting reflexes at that timepoint. **(C)** USV data split by sex and treatment. No sex differences were observed in response to treatment in either USV outcomes or developmental milestones (Fig. S2C; Table S1). These data represented as mean +/-SEM, *=p<0.05. USVs/Developmental Milestones (**MAR**; N=48, **Control**; N=40), Social Dyad (**MAR**; N=24; **Control**; N=20).


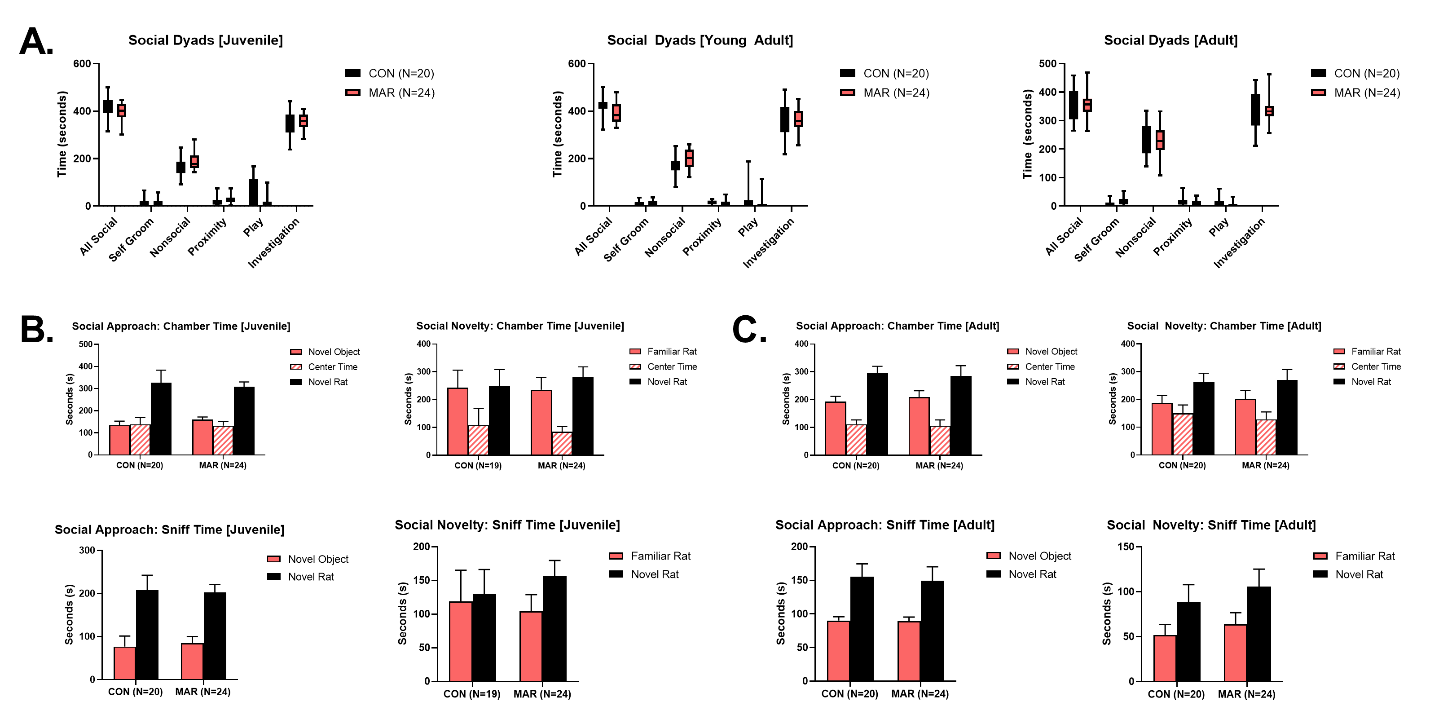


**Figure S3.** **Social Dyads, Three-Chamber Sociability and Social Novelty Test.**

The total duration spent engaging in social investigation in proximity to the stimulus rat, playing, self-grooming, and engaging in nonsocial activities presented at juvenile, young adult and adult timepoints **(A)**. The “All Social” metric includes proximity, play, and investigation time. Error bars show minimum to maximum values. MAR treated rats spent less time engaging in all social behaviors at all 3 timepoints (p=0.023) but spent more time in proximity to the stimulus rat at juvenile age (p=0.016). No treatment differences were observed in any outcome measures obtained from juvenile **(B)** and adult **(C)** offspring during the sociability phase (left panels) and the social novelty phase (right panels). Data are represented as mean + 95% CIs. Social dyad and social approach (**MAR**; N=24; **Control**; N=20).


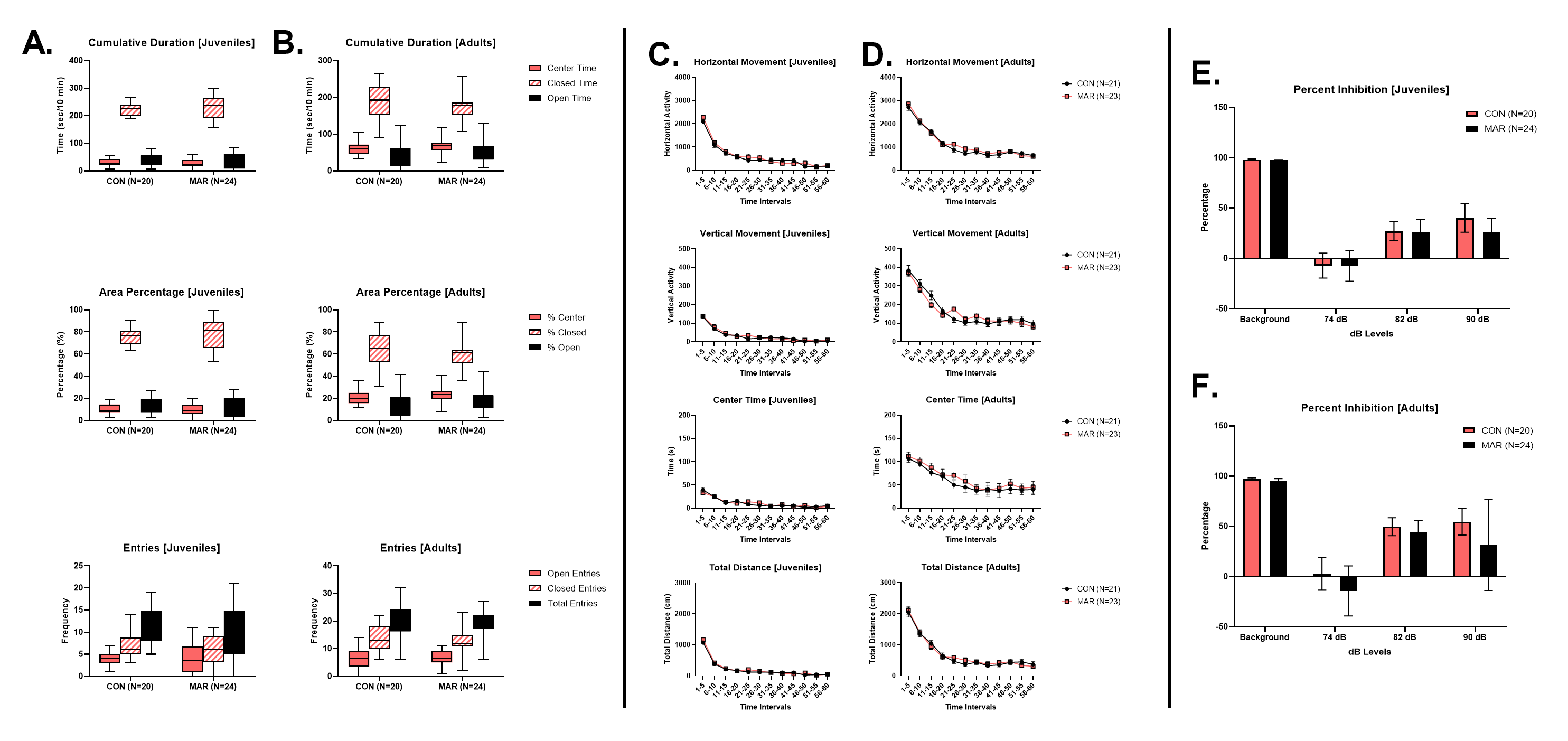


**Figure S4.** **Elevated Plus Maze, Open Field and PPI.** No treatment differences were observed in any outcome measures obtained from elevated plus maze (A-B), open field (C-D) or PPI (E-F). In the elevated plus maze, both MAR and CON exhibited species-typical anxiety-like behaviors as indexed by the total time in each arm, percentage in each arm, and entries to each arm for both juvenile (A) and adult (B). Error bars show minimum and maximum values. In the open field maze, both MAR and CON rats exhibited normal exploratory locomotion as indexed by total time spent moving horizontally and vertically, time spent in the center, and distance traveled are shown in 5-minute increments for both juvenile (C) and adult (D) animals. Species-typical inhibition of acoustic startle seen in MAR and CON offspring indicates intact sensorimotor gating as indexed by the percent inhibition in the PPI task is shown for juvenile (E) and adult (F) rats with error bars as the standard error of the mean. All tests (**MAR**; N=24; **Control**; N=20).

**Magnetic Resonance Imaging and Spectroscopy Results**


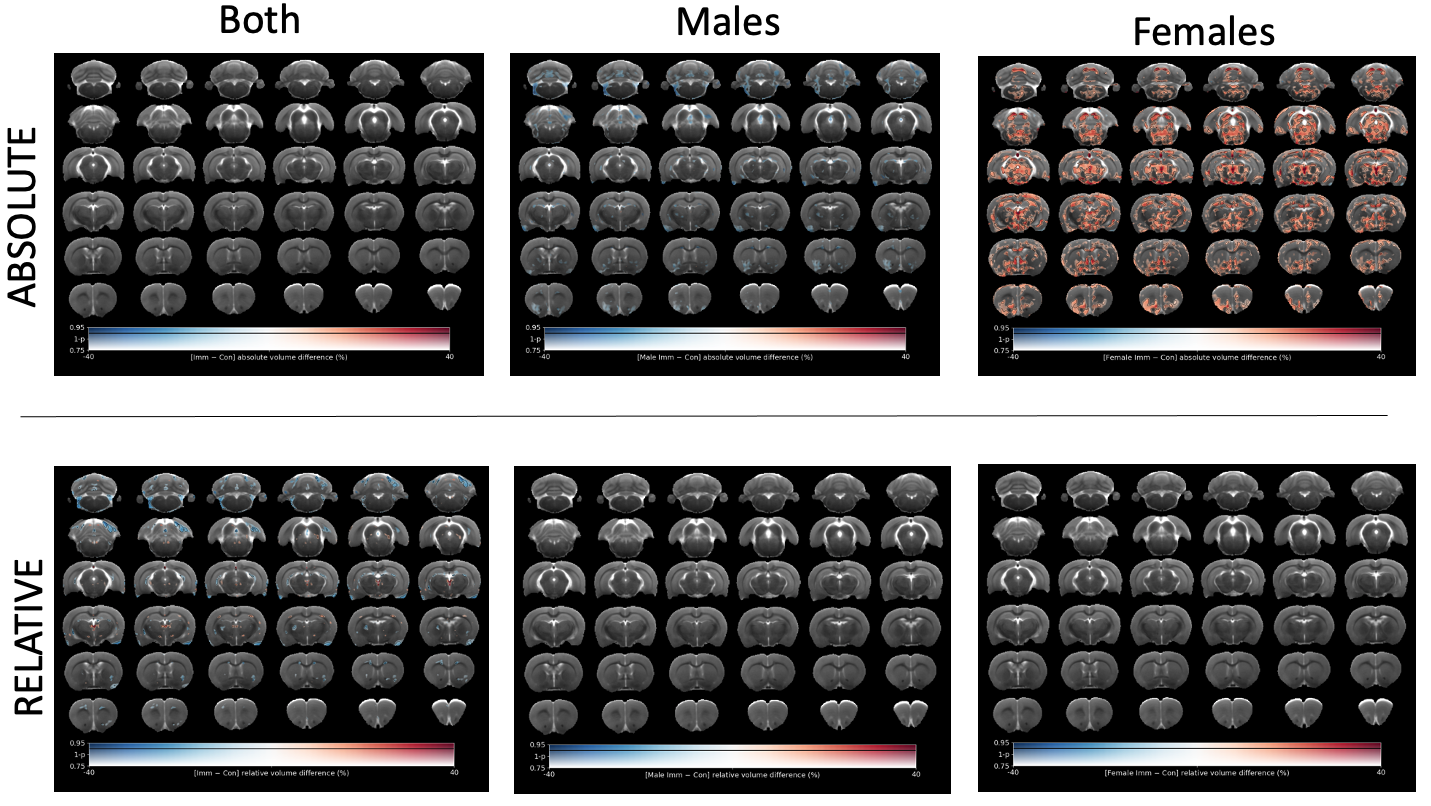


**Figure S5. Voxel-wise analysis of brain volume.** MRI images depicting voxel-wise data as a heatmap of the percent difference in absolute or relative volume between MAR-ASD (Imm) and control (Con) offspring. Split by sex or combined in the “both” condition. Red represents an increased volume difference while blue represents a volumetric decrease. Areas highlighted by contour represent clusters of voxels that passed 5% FDR correction. Uncorrected p-values are colored areas shown without contour.

**
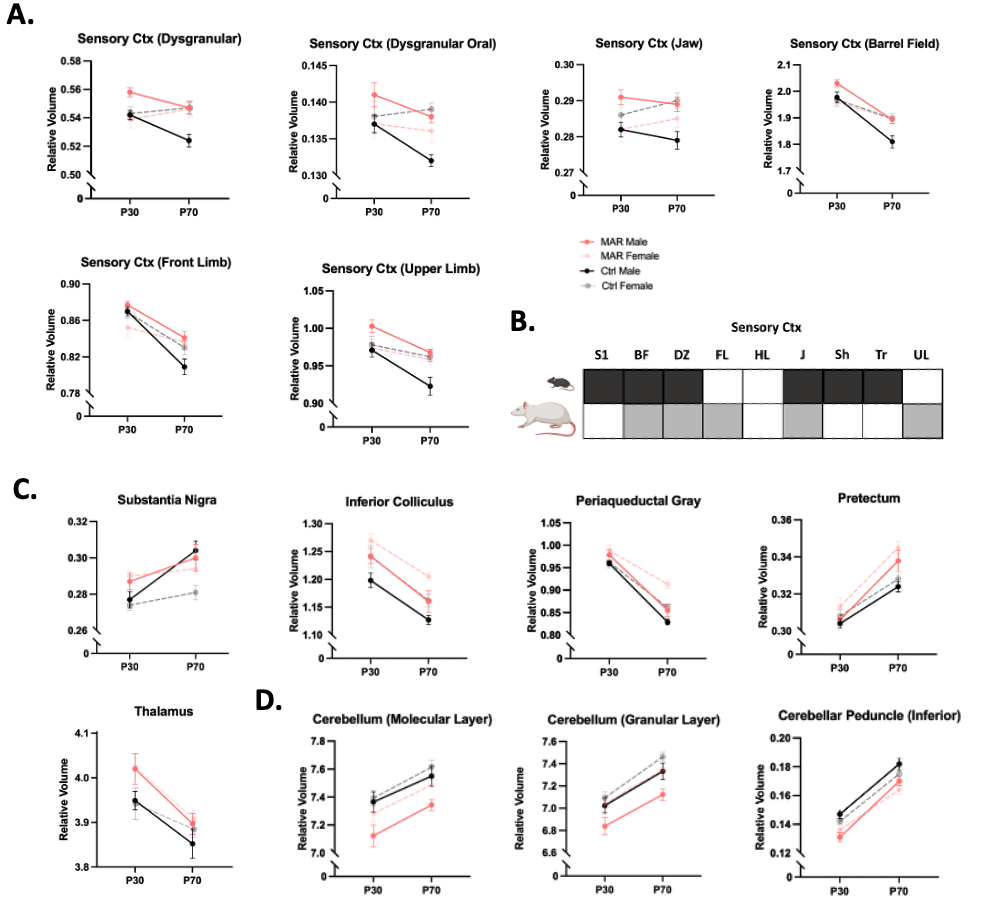
**

**Figure S6**. **Relative regional volumetric differences by time, sex, and treatment.** Regions determined to be significantly different by treatment in the sensory cortex **(A)**, midbrain **(C)**, and cerebellum **(D)**. **(B)** Comparison of regions affected by treatment within the sensory cortex between rats and mice (data taken from Bruce et al; 2021). Grey represents regions significantly different by treatment in rats and passing FDR correction (p<0.05, q<0.05). Black represents regions in MAR-ASD mice that passed significance testing but not multiple comparison testing (p<0.05, q>0.05).


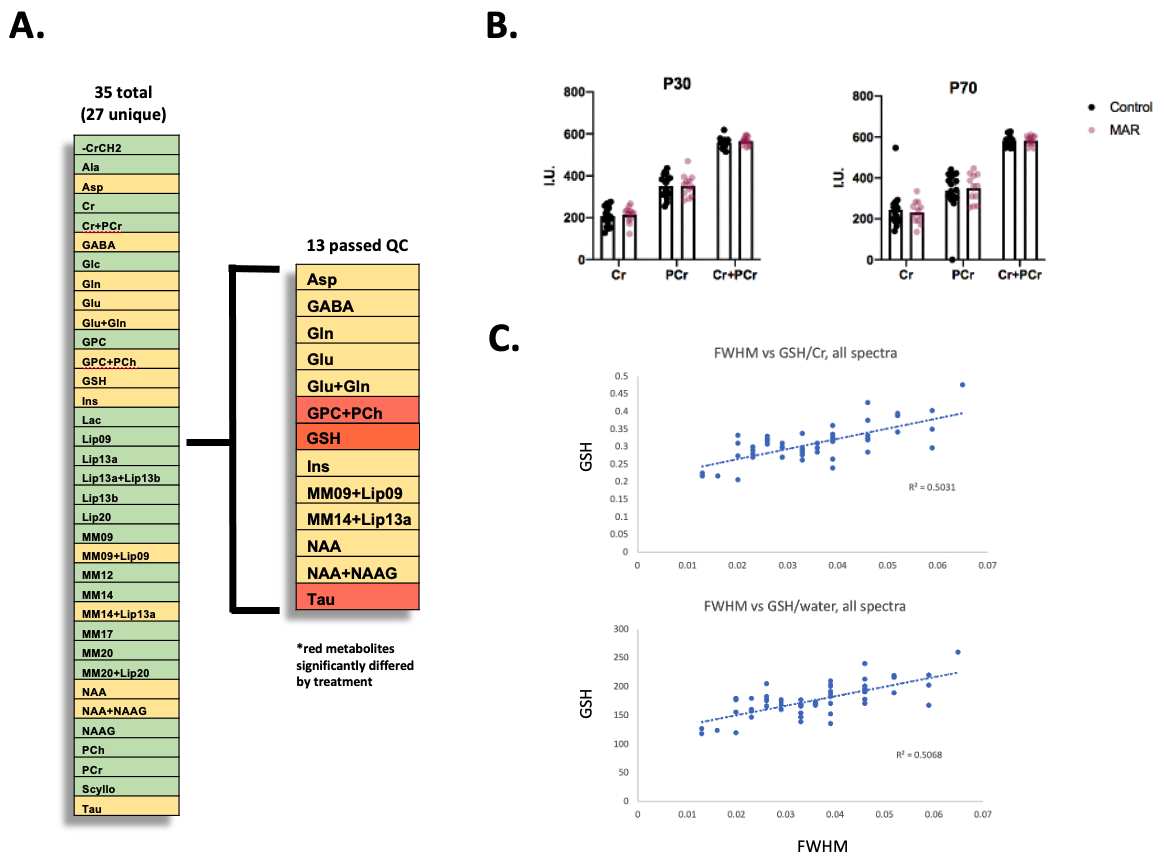


**Figure S7**. **Additional MRS metabolite data. (A)** Visual representation of total metabolites and quality control (QC) filtering. **(B)** Water-scaled creatine (Cr), phosphocreatine (PCr), or Cr + PCr values plotted by treatment group. Data expressed as mean +/- SEM. **(C)** Scatterplot of the correlation between glutathione (GSH) levels and full width half maximum (FWHM), shown normalized against creatine or water on separate graphs. R2 included as linear fit value.

1. M. Yang, J. L. Silverman, J. N. Crawley, Automated Three‐Chambered Social Approach Task for Mice. *Current Protocols in Neuroscience* **56**, (2011).

2. K. M. Ku, R. K. Weir, J. L. Silverman, R. F. Berman, M. D. Bauman, Behavioral Phenotyping of Juvenile Long-Evans and Sprague-Dawley Rats: Implications for Preclinical Models of Autism Spectrum Disorders. *PLoS One* **11**, e0158150 (2016).

3. R Core Team. (R Foundation for Statistical Computing, Vienna, Austria, 2022).

4. S. G. Self, K.-Y. Liang, Asymptotic Properties of Maximum Likelihood Estimators and Likelihood Ratio Tests under Nonstandard Conditions. *Journal of the American Statistical Association* **82**, 605-610 (1987).

5. M. K. Olsen, J. L. Schafer, A Two-Part Random-Effects Model for Semicontinuous Longitudinal Data. *Journal of the American Statistical Association* **96**, 730-745 (2001).

6. J. Hennig, A. Nauerth, H. Friedburg, RARE imaging: A fast imaging method for clinical MR. *Magnetic Resonance in Medicine* **3**, 823-833 (1986).

7. P. A. Bottomley, Spatial Localization in NMR Spectroscopy in Vivo. *Annals of the New York Academy of Sciences* **508**, 333-348 (1987).

8. I. Tkác, Z. Starcuk, I. Y. Choi, R. Gruetter, In vivo 1H NMR spectroscopy of rat brain at 1 ms echo time. *Magn Reson Med* **41**, 649-656 (1999).

9. M. Jenkinson, C. F. Beckmann, T. E. J. Behrens, M. W. Woolrich, S. M. Smith, FSL. *NeuroImage* **62**, 782-790 (2012).

10. B. B. Avants *et al.*, A reproducible evaluation of ANTs similarity metric performance in brain image registration. *NeuroImage* **54**, 2033-2044 (2011).

11. T. C Wood, QUIT: QUantitative Imaging Tools. *Journal of Open Source Software* **3**, 656 (2018).

12. N. J. Tustison *et al.*, N4ITK: Improved N3 Bias Correction. *IEEE Transactions on Medical Imaging* **29**, 1310-1320 (2010).

13. B. B. Avants *et al.*, The optimal template effect in hippocampus studies of diseased populations. *NeuroImage* **49**, 2457-2466 (2010).

14. I. Oguz, H. Zhang, A. Rumple, M. Sonka, RATS: Rapid Automatic Tissue Segmentation in rodent brain MRI. *Journal of Neuroscience Methods* **221**, 175-182 (2014).

15. W. R. Crum *et al.*, Evolution of structural abnormalities in the rat brain following in utero exposure to maternal immune activation: A longitudinal in vivo MRI study. *Brain, behavior, and immunity* **63**, 50-59 (2017).

16. W.-L. Kuan *et al.*, Systemic α-synuclein injection triggers selective neuronal pathology as seen in patients with Parkinson’s disease. *Molecular Psychiatry* **26**, 556-567 (2021).

17. M. H. Buonocore, R. J. Maddock, Magnetic resonance spectroscopy of the brain: a review of physical principles and technical methods. *Rev Neurosci* **26**, 609-632 (2015).
